# Genetic restriction of antigen-presentation dictates allergic sensitization and disease in humanized mice

**DOI:** 10.1016/j.ebiom.2018.04.001

**Published:** 2018-04-05

**Authors:** Alina Neunkirchner, Bernhard Kratzer, Cordula Köhler, Ursula Smole, Lukas F. Mager, Klaus G. Schmetterer, Doris Trapin, Victoria Leb-Reichl, Edward Rosloniec, Ronald Naumann, Lukas Kenner, Beatrice Jahn-Schmid, Barbara Bohle, Rudolf Valenta, Winfried F. Pickl

**Affiliations:** a Christian Doppler Laboratory for Immunomodulation, 1090 Vienna, Austria; b Institute of Immunology, Medical University of Vienna, 1090 Vienna, Austria; c Department of Laboratory Medicine, Medical University of Vienna, 1090 Vienna, Austria; d Department of Medicine, University of Tennessee Health Science Center, Memphis, 38163, TN, USA; e Memphis Veterans Affairs Medical Center, 38104, TN, USA; f Department of Pathology, University of Tennessee Health Science Center, Memphis, 38163, TN, USA; g Max Planck Institute for Molecular Cell Biology and Genetics, 01307 Dresden, Germany; h Department of Laboratory Animal Pathology, Medical University of Vienna, 1090 Vienna, Austria; i Department of Laboratory Animal Pathology, University of Veterinary Medicine Vienna, 1210 Vienna, Austria; j Ludwig Boltzmann Institute for Cancer Research, Vienna, Austria; k Department of Pathophysiology and Allergy Research, Medical University of Vienna, 1090 Vienna, Austria

**Keywords:** Sensitization, Airway hyperreactivity, T regulatory cells, IL-2-αIL-2 complexes, Allergen-specific TCR, Tolerance, Aeroallergen, Mugwort allergy

## Abstract

**Background:**

Immunoglobulin(Ig)E-associated allergies result from misguided immune responses against innocuous antigens. CD4^+^ T lymphocytes are critical for initiating and perpetuating that process, yet the crucial factors determining whether an individual becomes sensitized towards a given allergen remain largely unknown.

**Objective:**

To determine the key factors for sensitization and allergy towards a given allergen.

**Methods:**

We here created a novel human T cell receptor(TCR) and human leucocyte antigen (HLA)-DR1 (TCR-DR1) transgenic mouse model of asthma, based on the human-relevant major mugwort (*Artemisia vulgaris*) pollen allergen Art v 1 to examine the critical factors for sensitization and allergy upon natural allergen exposure *via* the airways in the absence of systemic priming and adjuvants.

**Results:**

Acute allergen exposure led to IgE-independent airway hyperreactivity (AHR) and T helper(Th)2-prone lung inflammation in TCR-DR1, but not DR1, TCR or wildtype (WT) control mice, that was alleviated by prophylactic interleukin(IL)-2-αIL-2 mAb complex-induced expansion of Tregs. Chronic allergen exposure sensitized one third of single DR1 transgenic mice, however, without impacting on lung function. Similar treatment led to AHR and Th2-driven lung pathology in >90% of TCR-DR1 mice. Prophylactic and therapeutic expansion of Tregs with IL-2-αIL-2 mAb complexes blocked the generation and boosting of allergen-specific IgE associated with chronic allergen exposure.

**Conclusions:**

We identify genetic restriction of allergen presentation as primary factor dictating allergic sensitization and disease against the major pollen allergen from the weed mugwort, which frequently causes sensitization and disease in humans. Furthermore, we demonstrate the importance of the balance between allergen-specific T effector and Treg cells for modulating allergic immune responses.

## Highlights

• Experiments in humanized mice identify genetic restriction of antigen-presentation as primary factor for allergies
• IL-2-αIL-2 complex induced Treg block sensitization and alleviate allergic disease
• Humanized TCR-DR1 allergy mice will help to identify and evaluate novel prophylactic and therapeutic allergy treatments

Humanized biological model systems are essential for the better understanding of the pathomechanisms operative in complex diseases such as allergies. Allergies target multiple aspects (cellular and humoral) of the human immune system and manifest themselves in target organs heavily exposed to the environment, *i.e.,* the respiratory tract, the gut and the skin. Although allergies affect >30% of individuals in our societies, the primary causes for sensitization and development of full-blown disease remain enigmatic. The here obtained results suggest that genetic restriction of allergen-presentation is the primary factor dictating sensitization and development of disease, especially when the allergen is administered in the “most natural” way, *i.e., via* the airways (for aeroallergens) in the absence of systemic priming and adjuvants.

## 1 Introduction

Immunoglobulin(Ig)E-associated allergic diseases are characterized by an aberrant immune response to usually innocuous environmental antigens (Larche et al., 2006). While major effector mechanisms of the disease are triggered by allergen-specific IgE antibodies, effector T lymphocytes play a pivotal role in the initiation and propagation of the allergic phenotype (Romagnani, 2004; Valenta et al., 2018). Apart from the recently discovered type 2 innate lymphoid cells (ILC2) (Maggi et al., 2017), CD4^+^ T helper cells represent the main source of interleukins (IL)-4 and IL-13, which promote immunoglobulin class switching towards IgE (Larche et al., 2006). In addition, results from clinical studies clearly demonstrated that T cells also play a major role in late-phase and chronic allergic reactions contributing to organ pathology in the airways, skin and gastrointestinal tract (Haselden et al., 1999; Karlsson et al., 2004; Werfel, 2009).

One major question is why certain individuals develop an allergic sensitization towards certain allergens. There are at least three mutually not exclusive hypotheses to answer this question: First, it is possible that certain individuals are genetically prone to preferentially recognize certain allergens. In fact, early studies in patient populations suffering from allergy to pollen (ambrosia, birch, mugwort), animal dander (cat) and mold (*Alternaria*) provided evidence that allergen-specific IgE production could be MHC-restricted (Fischer et al., 1992; Jahn-Schmid et al., 2005; Marsh et al., 1982; Young et al., 1994). This was confirmed when T helper cell clones from allergic patients were isolated and the existence of an allergen-specific genetic restriction of the allergen-specific immune response was demonstrated. For example, Th cell clones specific for the major mugwort (*Artemisia vulgaris*) pollen allergen Art v 1 were found to recognize one major T cell epitope, *i.e.,* Art v 1_25–36_, in the context of a dominant MHCII allele, *i.e.,* HLA-DR1 (Jahn-Schmid et al., 2005; Jahn-Schmid et al., 2002).

The second possibility why certain subjects develop allergy towards a given allergen would be an imbalance between effector and regulatory T cell responses towards the allergen. A study analyzing the frequency of IL-4 producing CD4^+^ T effector cells (Teff) and IL-10-producing T regulatory cells (Treg) in allergic and non-allergic subjects suggested that allergic subjects present with higher numbers of IL-4-producing CD4^+^ effector cells whereas IL-10-producing allergen-specific Tregs are increased in non-allergic subjects (Akdis et al., 2004). Since it was then demonstrated that CD4^+^CD25^high^Foxp3^+^ allergen-specific Treg cells are present and functionally active in both non-atopic and atopic individuals the question regarding the specific contributions of allergen-specific CD4^+^ effector cells and Tregs in the regulation of the allergen-specific IgE response arises. In fact, it is well established that extrathymically induced Treg subsets but also Tregs engineered by overexpression of the transcription factor *FOXP3* are extremely potent in controlling T cellular immune responses against environmental antigens including allergens (Schmetterer et al., 2011a, b; Shevach and Thornton, 2014; Verhagen et al., 2015). Moreover, expansion of CD4^+^ Treg using immune-complexes of IL-2 and anti-IL-2 antibodies, can be used to treat hypersensitivity diseases but also transplant rejection in experimental settings (Shevach, 2012; Webster et al., 2009).

Recently, another provocative possibility for developing allergy against a given allergen was introduced. It was claimed that the intrinsic properties of allergens (Bacher et al., 2016) are pivotal for the development of tolerance *versus* allergy against aeroallergens. Specifically, it was suggested that allergens, which rapidly dissociate from inhaled particles (*e.g.*, pollen) and become soluble in aqueous solutions, escape Treg-mediated suppression and thus drive allergen-specific Teff responses and allergic sensitization (Bacher et al., 2016).

In order to investigate the three hypotheses for allergic sensitization and to decipher the contribution and interplay of i) MHC-dependent recognition of allergen-specific T cell epitopes, ii) the corresponding activation and expansion of allergen-specific Teff as well as Treg cells in the allergic sensitization process and in immune pathology and iii) the role of natural antigenic exposure, we established a unique humanized mouse model. This model is based on a human-relevant major pollen allergen and simultaneous expression of the corresponding human T cell receptor (TCR) and human leucocyte antigen (HLA) molecules. We selected mugwort allergy driven by the major mugwort pollen allergen of *Artemisia vulgaris*, Art v 1, as the human-relevant model system. Mugwort represents an important aeroallergen source growing in the European temperate climate zone, throughout North America and parts of Asia (Charpin et al., 1974; D'Amato et al., 1998; Wopfner et al., 2005). It is one of the main causes of hay fever and asthma in late summer and fall (Himly et al., 2003; Torio et al., 2003). Notably, sensitization to mugwort nearly exclusively depends on the major mugwort pollen allergen Art v 1 (in 95% of affected allergic individuals) and the presentation of its immunodominant T cell epitope, Art v 1_25–36_, which is highly restricted by HLA-DRA*01-HLA-DRB1*01 (Jahn-Schmid et al., 2002, 2005). Importantly, an unusually high odds-ratio of 8.45 (range 4–17) for the presence of the HLA-DRB1*01 allele and the probability of getting sensitized against mugwort (Art v 1) was observed by comparing mugwort allergic patients with healthy control populations (Jahn-Schmid et al., 2005). This represents the single strongest association at the T cell epitope level between the presence of an MHC class II allele and allergic sensitization.

For the generation of TCR tg mice we took advantage of a human mugwort-specific TCR which was cloned and functionally characterized by us previously (Leb et al., 2008). Using this TCR and HLA-DRA*01-HLA-DRB1*01 (DR1) transgenic mice (Rosloniec et al., 1997) we generated allergen-specific TCR-DR1 transgenic mice to investigate the contribution of allergen-specific MHCII as well as of allergen-specific CD4^+^ Teff and Treg to the initiation of allergen-specific sensitization. In addition, we studied the importance of Tregs for the prevention and treatment of allergy in this model.

## 2 Material and methods

### 2.1 Recombinant allergens and peptides and preparation of pollen extracts

Purified recombinant Art v 1.0101 and Bet v 1.0101 allergens were purchased from Biomay AG (Vienna, Austria). Immunodominant peptides from major mugwort (Art v 1_25–36_) and birch (Bet v 1_142–153_) pollen allergens were obtained from ProImmune (Oxford, UK).

*Preparation of pollen extracts: Artemisia vulgaris* pollen (Allergon AB, Engelholm, Sweden or Greer Laboratories, Lenoir, NC) were used for the preparation of aqueous mugwort pollen extracts according to standard procedures. Briefly, 10 g of mugwort-pollen were incubated in 100 ml of PBS (1×) by stirring at 4 °C overnight. After centrifugation at 52,000*g* at 4 °C for 60 min, the supernatants were filtered and subsequently dialyzed (Spretra/Por Dialysis Membrane, MWCO: 6–8000, Spectrum Laboratories, Rancho Dominues, CA) against 1× PBS for 48 h. The total protein concentration of the dialysate was determined by standard procedures (BCA-bicinchoninic acid protein Kit, Pierce, Rockford, IL). The lipopolysaccharide (LPS) content of the mugwort pollen extract was ≤0,024 U/mg. The extracts were lyophilized and aliquots were stored at −80 °C.

### 2.2 PCR amplification of TCR sequences

Amplification of TCR specific DNA sequences from the original T cell clone SSR20 was performed using the oligonucleotide primers 5′-CGC GGG CCC GGG AGG TCT TCT GTG ATT TCA ATA AGG A-3′ (sense) and 5′-CCC GCG GCG GCC GCC CCC ATG AGG ACT GCA TTT TG-3′ (antisense) for the α-chain and 5′-CGC GGG CTC GAG GTG CCT TTG CCC TGC CTG T-3′ (sense) 5′-CCC GCG CCG CGG ACA CCC AGC TCC TCC AGC-3′ (antisense) for the β-chain. Both PCR fragments (size: 653 bp and 809 bp, respectively) were digested with appropriate restriction enzymes (α-chain: *Xma I*/*Not I*; β-chain: *Xho I*/*Sac II,* New England Biolabs, Ipswich, MA) and cloned into the pUC19 derived pBluescript SK^+^ vector (Stratagene, Heidelberg, Germany).

### 2.3 Generation of TCR transgenic mice

To generate TCR tg mice, rearranged V(D)J regions of the TCR from the human Art v 1-specific and HLA-DRB1*01:01-restricted TH0 cell clone SSR20, as described previously (Jahn-Schmid et al., 2005; Leb et al., 2008), were cloned into the TCR cassette vectors pTαcass and pTβcass (kindly provided by Dr. Diane Mathis, Harvard Medical School, Boston, MA (Kouskoff et al., 1995)). Variable TCR regions were amplified by PCR from genomic DNA of the original T cell clone SSR20 and cloned into the pUC 19 derived vector pBluescript SK^+^ (Stratagene, Heidelberg, Germany) for sequence verification. Upon successful transient expression in HEK-293 cells along with the murine CD3 complex, pTαcass (*Sal* I) and pTβcass (*Kpn* I) vectors were linearized to remove prokaryotic vector sequences followed by microinjection into pronuclei of fertilized eggs of C57BL/6-J mice. Five founder mice were obtained, two of which revealed germline transmission of transgenes. Offspring were analyzed by PCR of tail biopsy DNAs using TCRα or TCRβ clonotype-specific PCR and applying the above-described primer devoid of restriction sites and 5′-clamp sequences. Mice from the positive founder line A003, showing the highest TCR expression levels on CD4^+^ T cells, were crossed with B10.M-DR1^d1AB1-Ea^ mice and backcrossed onto the C57BL/6-J background for ≥10 generations before experimental use and were bred towards homozygosity. Corresponding mouse lines were termed *TCR-DR1* for TCRα/β x B10.M-DR1^d1AB1-Ea^ tg mice, *TCR* for TCRα-β tg mice, *DR1* for C57BL6-J-DR1^d1AB1-Ea^ tg mice and WT for wild type C57BL6-J mice. All experimental procedures were reviewed and approved by the Institutional Review Board of the Medical University of Vienna and approved by the Federal Ministry of Science, Austria (BMWF-66.009 0118-II 3b 2012).

### 2.4 Mice and animal experimental procedures

Homozygous HLA-DR1 mice (B10.M-DR1^d1AB1-Ea^) (Rosloniec et al., 1997) were obtained from the Veterans Affairs Hospital, Memphis, TN. Wild type C57BL6J (Charles River, Sulzfeld, Germany) and the corresponding single TCR or DR1 and double TCR-DR1 transgenic mouse strains (all back-crossed at least 10–12 times to C57BL6J) were cohoused in a conventional animal facility at the Institute of Immunology (Medical University of Vienna, Vienna, Austria). Age-matched female mice (6–10 weeks old) were used for experiments. All mice received food and water *ad libitum*. Sentinel mice were screened for and found to be free of mouse pathogenic viruses, bacteria and parasites according to FELASA 2014 recommendations (Mahler Convenor et al., 2014).

*Acute (short-term) sensitization protocol:* Mice were sensitized and challenged *via* the airways with nebulized aqueous mugwort pollen extract (1%) for 16 min on three consecutive days.

*Chronic (long-term) sensitization protocol:* Mice were sensitized and challenged by intranasal (i.n.) administration of 20 μl of a 1.5% aqueous mugwort pollen extract solution once every two weeks for up to 8 weeks. Blood samples were collected prior to the first immunization and 10 days after each immunization by tail vein incision. Blood samples were taken one day after the last exposure and serum stored for each mouse individually at −20 °C.

*Determination of AHR:* The lung function in the four groups of mice (WT, DR1, TCR or TCR-DR1) was analyzed by unrestrained whole body plethysmography (Buxco, Winchester, UK) or, alternatively, by invasive determination of lung resistance (Finepoint, Buxco) on tracheostomized and i.p. anesthetized mice (100 mg/kg Ketamine (Ketanest, Pfizer, Vienna, Austria) and 5 mg/kg Xylazine (Rompun, Bayer, Leverkusen, Germany)), respectively, which were maintained on a 37 °C tempered table for the whole duration of the procedure. During both procedures mice were challenged with increasing doses of methacholine (range 1.5–12.5 mg/ml in phosphate buffered saline (PBS)) (Hamelmann et al., 1997) or alternatively 1% mugwort-pollen extract solution as indicated in the respective figure. PBS only was used as control solution.

*Footpad swelling assays and determination of delayed type hypersensitivity:* Anesthetized animals received an intradermal injection into the right hind footpad with 25 μl of allergen extract preparation (containing 375 μg whole protein content) using a 1 ml syringe equipped with a hypodermic needle (26G, 0.45 × 10 mm, Becton Dickinson, Palo Alto, CA), while the contralateral hind footpad was challenged with PBS for control purposes. After 24, 48, 72 and 96 h the appearance of the individual injection sites were inspected and documented. Thickness of the footpads before and after immunization was measured using a digital thickness gauge and Δ-levels of footpad swelling were determined as follows: Footpad swelling (mm) = footpad thickness after allergen provocation - footpad thickness before allergen provocation.

*In vivo expansion of Treg by IL-2-α-IL-2 complexes:* IL-2 (1 μg, Peprotec, London, UK) and anti-IL-2 antibody JES6-1 (5 μg, Life Tech Austria, Vienna, Austria) were pre-incubated *in vitro* to ensure complex formation. Subsequently, TCR-DR1 mice were i.p. injected with 6 μg of this complex on the indicated three consecutive days (Boyman et al., 2006; Webster et al., 2009).

### 2.5 Whole blood and tissue collection

Whole blood samples were collected by incision of the tail vein into 10 U/ml heparin containing tubes. Spleens, lymph nodes and thymi of animals were obtained upon anatomical dissection according to standard procedures from animals sacrificed under isoflurane anesthesia followed by cervical dislocation. Briefly, tissues were homogenized in medium by mincing organs cut into 5 mm pieces with the pestle of a 10 ml syringe followed by sieving through a 70 μm nylon cell strainer (Becton Dickinson) to obtain single cell suspensions, which were washed subsequently in IMDM (GE Healthcare, Pasching, Austria) plus 10% FCS, 2 mM l-glutamine, essential amino acids, 0.1 mM 2-mercaptoethanol, 1 mM sodium pyruvate and 25 mM HEPES (pH 7.3, Sigma Chemicals, St. Louis, MO) at 200*g* for 10 min. Erythrocytes were removed by incubation of cell suspensions in ammonium chloride lysis buffer (155 mM ammonium chloride, 10 mM potassium hydrogen carbonate, 0.1 mM EDTA at pH 7.40, Sigma Chemicals) at room temperature for 5 min. Subsequently, cells were washed twice in IMDM at 200*g* for 10 min and adjusted to a concentration of 1 × 10^7^/ml in medium. Lung homogenates were prepared according to standard procedures. Briefly, lungs were collected, chopped into 5 mm pieces and incubated with digestion solution containing 1.8 μg/ml collagenase and 40 μg/ml DNase (Sigma Chemicals) at 37 °C for 60 min. Then, cells were disaggregated and filtered through a 70 μm nylon cell strainer (Becton Dickinson). The filtrate was centrifuged at 500 ×*g* at 4 °C for 5 min. The cell pellet was re-suspended in ammonium-chloride lysis buffer, incubated at room temperature for 5 min, washed once in 1× IMDM before subjection to flow cytometric analyses.

### 2.6 Flow cytometric analyses of leukocyte populations in peripheral blood, spleens, thymi and lung tissues

*Cell surface staining:* Flow cytometry on peripheral blood (PB), spleens, thymi and lung tissues was performed according to standard procedures (Cossarizza et al., 2017) using the binding reagents listed in Table S2. Briefly, 20 μl aliquots of anticoagulated whole blood were incubated with the indicated mAb-combinations (20 μg/ml) at room temperature for 20 min. After addition of 100 μl lysis solution (An der Grub, Kaumberg, Austria) and incubation at room temperature for 10 min, 5 ml of ddH_2_0 was added, incubated for 5 min followed by centrifugation at 500*g* for 5 min. Subsequently, supernatants were discarded and the samples were analyzed by flow cytometry. Splenocytes and thymocytes were harvested as described above, re-suspended in 1 × 10^6^ cells/50 μl of PBS supplemented with 0.5% bovine serum albumin (BSA) and incubated with 20 μl of the indicated mAb combinations on ice for 30 min. Afterwards, cells were washed once with PBS plus 0.5% BSA and submitted to flow cytometric analyses. Data were obtained exclusively from viable cells by using appropriate forward and side scatter gating.

*Intracellular cytokine staining:* For intracellular cytokine staining, lung homogenates were prepared as described in the respective sections. 1 × 10^6^ cells were incubated in the presence of 125 ng/ml ionomycin and 100 nM PMA (Sigma Chemicals) overnight and for the last 6 h supplemented additionally with 2 μM monensin plus 3 μg/ml brefeldin A (eBioscience, San Diego, CA). Subsequently, cells were fixed according to the manufacturers protocol (eBioscience) and stained with the antibodies listed in Table S1.

*Intracellular Foxp3 staining:* The staining of Foxp3 expressing cells in lung homogenates and in peripheral blood was performed according to the Foxp3 staining set (eBioscience). Briefly, cells were collected as described above and first stained for surface antigens. After washing, fixation was performed at room temperature for at least 1 h or at 4 °C overnight. Subsequently, staining for Foxp3 was performed in permeabilization buffer at room temperature for 30 min. The different antibodies listed in Table S1 were used for the analyses of Tregs. All flow cytometric analyses were performed on a Fortessa LSR-II flow cytometer (Becton Dickinson) and data were analyzed with the Flow Jo v 10.2. software package (Tree Star Inc., Ashland, OR).

### 2.7 T cell proliferation and suppression and cytokine determination assays

*T cell proliferation assays:* Single cell suspensions of splenocytes (2 × 10^5^/well) were incubated in 96-well flat bottom plates with Art v 1_25–36_ peptide, full-length recombinant Art v 1 protein or Bet v 1_142–153_ peptide used as an independent specificity control. Concentrations of Art v 1 protein and peptide ranged from 0.125 μmol/l to 0.001 μmol/l and 1.5 μmol/l to 0.01 μmol/l, respectively. After 72 h cells were pulsed with methyl-[^3^H]thymidine (1 μCi/well) for 18 h and T cell proliferation was quantified on a Betaplate Counter (Packard, Meriden, CT). Similarly*,* splenocytes of mice were labeled with cell proliferation dye-V450 (eBioscience, 5 μmol/l) stimulated with Art v 1_25–36_ peptide, Bet v 1_142–153_ peptide, PMA/ionomycin or medium alone and analyzed by flow cytometry after 72–96 h using CD3, CD4, CD8 and TCRVβ18 mAB. Division indices of CD3^+^CD4^+^ T cells were calculated accordingly (Asquith et al., 2006) using the formula n = [log ((MFI of unstimulated population)/(MFI of stimulated population))]/log [2], which estimates the mean number of cell divisions between the unstimulated and the simulated cultures.

*T cell suppression assays:* In TCR-DR1 mice, Tregs were induced as described previously (Boyman et al., 2006; Webster et al., 2009). Briefly, mice received 1 μg of rIL-2 and 5 μg of αIL-2 antibody JES6–1 (Life Tech Austria, Vienna, Austria) on days 1, 2, and 3. On day 6, LN were isolated and Tregs were first enriched with a magnetic bead-based negative selection kit for CD4^+^ T cells (Miltenyi, Bergisch Gladbach, Germany). Subsequently, Tregs were FACS sorted to high purity (FACS Aria, Becton Dickinson) using the CD3^+^CD4^+^CD25^bright^ phenotype as marker. For the isolation of effector CD4^+^ T cells, LN of untreated age and sex matched TCR-DR1 mice were isolated, T cells were enriched by magnetic bead-based negative selection (Miltenyi) and purified by FACS sorting using CD3^+^CD4^+^CD25^−^ as the selecting phenotype. In the suppression assay, CD3^+^CD4^+^CD25^−^ Teff cells (5 × 10^4^, CPD eFluor® 450 labeled) were co-incubated with 1 × 10^4^ irradiated (90 Gy) DC2.4-DR1^+^ cells (Shen et al., 1997) as APCs the indicated ratios of Treg cells plus 1 μM of Art v 1_25–36_ peptide as antigen specific stimulus in quadruplicates. The division index calculated according to the Flow Jo software package v10.3. (Treestar, Ashland, OR) was taken as a measure for the degree of T cellular proliferation.

*Cytokine determination assays:* Supernatants of splenocyte cultures incubated with the indicated stimuli (see above) were harvested at various time points after initiation of culture (24–96 h) and subjected to multiplex cytokine analyses. Determination of secreted cytokines was performed using the FlowCytomix Multiple Analyte Detection System (eBioscience), a bead based multiplex immune assay for flow cytometry according to the manufacturer's recommendations. Beads were analyzed on a FACScalibur cytometer (Becton Dickinson). Alternatively, cytokine measurements were performed by multiplex analysis using the Luminex system (Luminex 100IS, Biomedica, Vienna, Austria) using the antibodies listed in Table S1.

*Analysis of epithelial cell stimulation:* For stimulation with aqueous mugwort pollen extract, 3 × 10^4^ BEAS-2B epithelial cells (ATCC, Manassas, VA, USA) were seeded per well of a 96-well plate in BEBM base medium (Lonza, Allendale, NJ) and grown until they reached confluence. Subsequently, cells were serum starved overnight before incubation with aqueous mugwort pollen extract (100 μg/ml) in medium for 30 min and 24 h. Cell culture supernatants were harvested after the indicated time points and analyzed for secreted IL-33 by ELISA (R&D Systems, Minneapolis, MN).

### 2.8 Determination of allergen-specific serum immunoglobulins (ELISA)

ELISA was performed to determine Art v 1-specific Ig in the sera of the differently immunized mice. ELISA plates (NUNC maxisorb, Sigma Chemicals) were coated with 2 μg/ml of recombinant Art v 1 protein in carbonate buffer (pH 9.6) overnight. Plates were washed with PBS plus 0.05% Tween (PT) two times and blocked with PT plus 1% BSA at room temperature for 2 *h. Sera* of immunized mice were diluted in PT plus 0.5% BSA 1:10 for IgE, 1:500 for IgG2 and 1:2000 for IgG1 determinations, respectively. Plates were incubated with 50 μl of diluted sera at 4 °C overnight and subsequently washed five times with PT. Specific Ig were detected by addition of 100 μl of monoclonal rat-anti-mouse Ig-subclass specific antibodies (BD Pharmingen, Palo Alto, CA) diluted 1:500 in PT 0.5% BSA at 37 °C for 3 h. After 5 washes with PT 100 μl of 1:2000 diluted goat-anti-rat horseradish peroxidase coupled antibody (GE Healthcare) were added and incubated at 37 °C for 30 min and at 4 °C for 30 min. Finally, plates were washed 5 times with PT and incubated with 100 μl ABTS substrate (Sigma Chemicals, St. Louis, MO). Plates were developed at room temperature in the dark, and extinction (optical density 405 nm) was measured with an ELISA reader (Multiskan GO) equipped with the SkanIt software (ThermoFisher Scientific, Fremont, CA) after 1 h.

### 2.9 Lung histology, immunohistochemistry and analysis of bronchoalveolar lavage fluid (BALF)

*Lung histology:* Lungs were initially fixed in 10% PBS buffered formaldehyde (Rotifix, Lactan, Graz, Austria) for 6 h and kept in 4% buffered formaldehyde (Rotifix) for 1–2 days before paraffin embedding. Serial tissue sections (2 μm) were stained with hematoxylin and eosin (H&E) dye using standard protocols. Lung infiltration was scored as described previously (Wilson et al., 2007). Briefly, for the entire lung section: presence of perivascular inflammation, 1; inflammation around 3 or more bronchioles, 2; dense inflammatory foci 3 cells deep, 3; loss of lung architecture, 4.

*Immunohistochemistry:* Immunohistochemical staining was performed on 2 μm tissue sections. First, tissue sections were de-paraffinized and rehydrated. Briefly, paraffin was melted at 60 °C overnight, and washes in four descending ethanol series were performed (absolute, 95%, 70%, 50% ethanol). Subsequently, antigens were retrieved in Tris/EDTA buffer (1 M, 0.05 M, respectively, pH 9.0) using a pressure cooker method (2100 Retriever, Aptum Biologics Ltd., Southampton, United Kingdom) for 20 min, followed by cooling for 20 min. To reduce background staining, four blocking steps were performed. Firstly, the endogenous peroxidase activity was blocked using 3% (v/v) hydrogen peroxidase (Roth, Karlsruhe, Germany). Secondly, an avidin/biotin blocking reagent (Vector, Burlingame, CA) was used. Accordingly, slides were rinsed 3 times with 70 ml wash buffer (1× PBS without Ca^2+/^Mg^2+^) for 5 min. Subsequently, slides were incubated with one drop of avidin solution (Vector) for 10 min in a staining chamber, followed by one rinse in 70 ml wash buffer followed by incubation with one drop of biotin solution (Vector) at room temperature for 10 min. Thirdly, an anti-mouse kit (Empire Genomics, Buffalo, NY) was applied. Tissue sections were rinsed in 70 ml wash buffer for 5 min, placed in a staining chamber and incubated with one drop of the mouse block at room temperature for 1 h. Finally, tissue sections were rinsed in 70 ml wash buffer for 5 min and incubated with 50 μl goat serum, diluted 1:10 in wash buffer, at room temperature for 10 min. Goat serum was removed by tilting and samples were incubated with optimized concentrations of primary antibodies at 4 °C in the dark overnight. CD3^+^ T cells were stained with the rabbit CD3 mAb (clone SP7, 1:300 diluted, ThermoFisher Scientific), CD4^+^ T cells were stained with the rat CD4 mAb (clone 4SM95, 1:100 diluted, eBioscience, San Diego, CA), B cells were stained with the rat mAb CD45R/B220 (clone RA3-6B2, 1:200 diluted, eBioscience, San Diego, CA), respectively. After rinsing tissue sections 3 times in 70 ml wash buffer for 10 min, samples were incubated with 50 μl of the respective secondary antibodies at room temperature in the dark for 30 min. For primary rat antibodies, a biotinylated secondary goat anti-rat IgG (#BA-9400, Vector), and for primary rabbit antibodies, a biotinylated secondary goat anti-rabbit IgG (#BA-1000, Vector) was used at a dilution of 1:300, respectively. Tissue sections were rinsed in 70 ml wash buffer for 10 min and samples were incubated with 50 μl avidin/biotin ABC complex (Vector), diluted 1:25 in wash buffer at room temperature for 45 min followed by 3 washing steps (10 min). Finally, bound antibodies were visualized by DAB immunostaining using the Metal Enhanced DAB Substrate Kit (ThermoFisher, Burlingame, CA).

*Analysis of bronchoalveolar lavage fluid (BALF) cells:* One day after the last challenge BALF was collected by flushing the airways two-times with 1 ml of ice-cold PBS (without Ca^2+^ and Mg^2+^). Cells were collected by centrifugation at 500*g* for 5 min. Total leukocyte cell numbers were determined and cytospin preparations (Cytospin 4 centrifuge, Thermofisher) of BALF cells were stained using a modified Wright-Giemsa stain (HematTek Stain Pack, Siemens, Erlangen, Germany) on a Hematek slide stainer (Siemens). Leukocyte subsets were determined by morphological criteria and routinely >200 cells were counted per cytospin slide by two individuals in a blinded manner by microscopy. In parallel, BALF cells were stained with the mAb panel as described (Table S2B). To prevent non-specific binding to Fc receptors the 2.4G2 blocking reagent (6 μg/ml, Becton Dickinson) was added to the mAb mix. The detailed cellular composition of BALF was determined on a Becton Dickinson Fortessa flow cytometer using FACS DIVA (Becton Dickinson) and FlowJo softwares (Treestar, Costa, Mesa, CA).

All microscopic slides were examined on a Nikon Eclipse E600 Fluorescence Microscope (Nikon Instruments, Amsterdam, Netherlands) equipped with an oil immersion 60×/1.40 objective and an 10×/0.30 objective. Pictures were taken by a Ds-Fi2 high definition color camera (Nikon Instruments), stored by the NIS-Element Viewer Software (Nikon Instruments) and processed with the Adobe Photoshop CS6 program (Adobe Systems, San Jose, CA).

### 2.10 Statistical analyses

Groups with similar variance were compared using a parametric test such as Student's *t*-test or one-way ANOVA followed by correction of alpha according to Bonferroni or Tukey using GraphPad version 6.0 (GraphPad Software Inc., La Jolla, CA). Otherwise, the Mann-Whitney *U* test or the Kruskal-Wallis test was performed, followed by Dunn's multiple comparison testing. Statistically significant values are indicated as *p < .05, **p < .01, ***p < .001, respectively.

## 3 Results

### 3.1 TCR-DR1-restricted transgenic mice develop functional TCR tg T lymphocytes and tg HLA-DR1^+^ antigen presenting cells

**Fig. 1:**
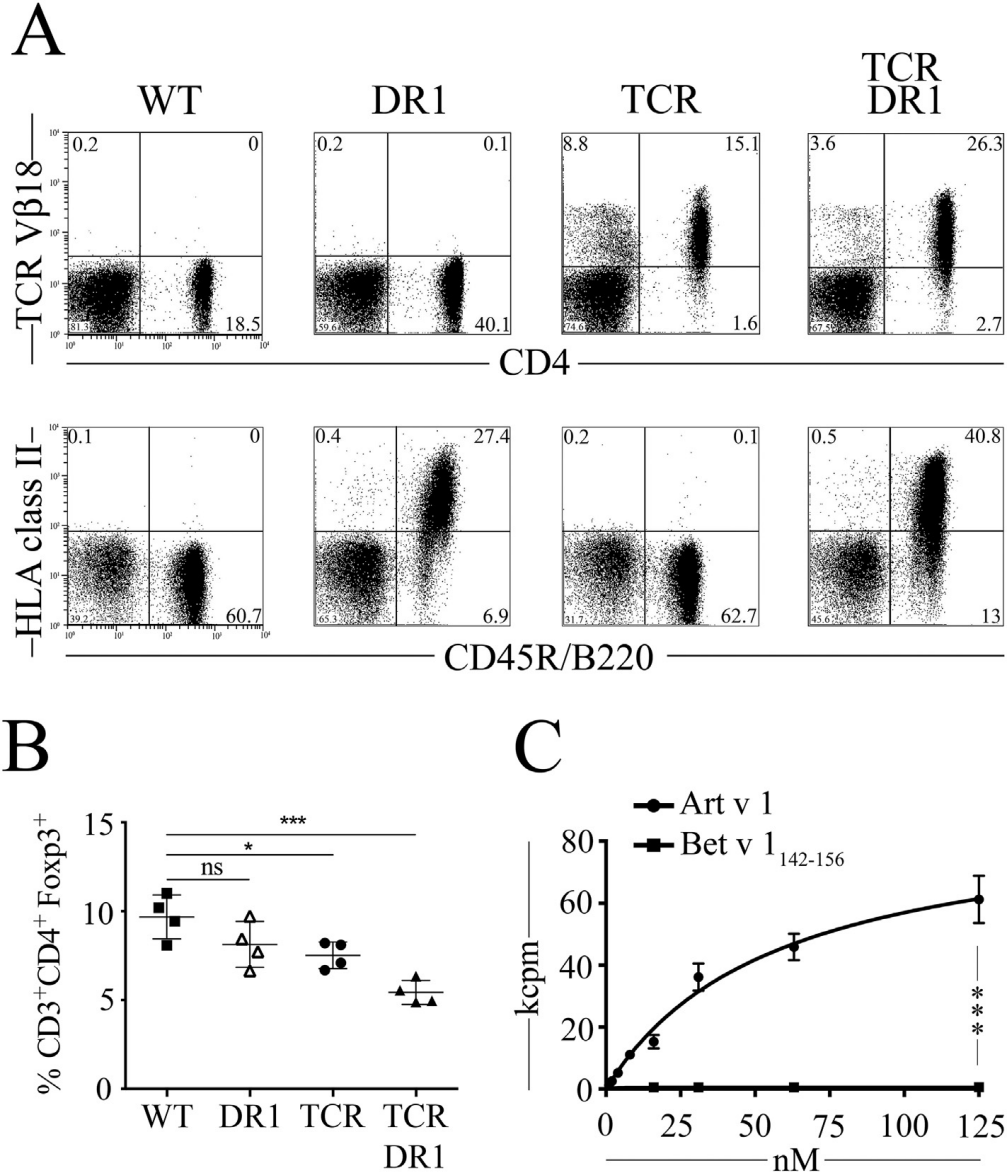
Expression and function of chimeric Art v 1-specific TCR and HLA-DR1. **A**, Flow cytometry analysis of TCR Vβ18 and HLA-DR1 expression on CD4^+^ and CD45R/B220^+^ PB lymphocytes isolated from WT, DR1, TCR and TCR-DR1 mice. Markers indicate negative control mAbs, numbers show percentage of cells. *n* = 4 per group of four analyses. **B,** Percentages of CD3^+^CD4^+^Foxp3^+^ cells in lymph nodes of WT, DR1, TCR and TCR-DR1 mice. Symbols represent individual mice. **C,** Dose dependent proliferation of TCR-DR1 splenocytes upon incubation with rArt v 1-protein, or Bet v 1_142–153_-peptide (negative control). kcpm, kilo counts per minute of incorporated [^3^H]-thymidine. Shown are representative SEMs of triplicate cultures of one out of four independent experiments. ***p < .001, Kruskal-Wallis test and Mann-Whitney-*U* test followed by *post hoc* Bonferroni correction.

We here established Art v 1-specific TCRαβ transgenic mice by grafting the human α- and β-chain variable domains of a previously characterized mugwort-specific TCR (Leb et al., 2008) onto mouse constant TCR regions (Fig. S1). The TCR tg founder line A003 was crossbred with HLA-DR1 mice (B10.M-DR1^d1AB1-Ea^) (Rosloniec et al., 1997) to obtain TCR-DR1 mice. TCR-DR1 mice have a life-span similar to WT mice and reveal no signs of immune pathology. Analyses of major immune cell subsets in WT, DR1, TCR and TCR-DR1 mouse lines revealed that CD3^+^CD4^+^ T cell and CD45R/B220^+^ B cell numbers did not differ between TCR-DR1 and WT mice in thymus, spleen or peripheral blood (PB) (Table S2). In contrast, TCR mice revealed lower CD3^+^CD4^+^ T cell numbers while DR1 mice presented with lower CD45R/B220^+^ B cell numbers compared to the other three strains of mice, respectively. Compared to WT mice, all three mouse strains have reduced numbers of CD8^+^ T cells. In PB, 62.1 ± 20.8% of CD3^+^CD4^+^ T cells expressed the transgenic TCR Vβ18-chain (Fig. 1**, A** and Table S2**)**, indicating proper selection of the Art v 1-specific TCR on the HLA-DRA*01:01-DRB1*01:01 background. Similarly, clear-cut HLA-DR1 expression was found on CD45R/B220^+^ B cells (Fig. 1A) and on CD11b^+^CD11c^+^ dendritic cells (Fig. S2A). Similar to previous findings in TCR model systems (Attridge and Walker, 2014), both TCR-DR1 and TCR mice present with lower relative numbers of CD3^+^CD4^+^Foxp3^+^ Treg cells in the periphery (lymph node (LN) and spleen) when compared to WT or DR1 mice (Fig. 1**, B** and **S3, A**). The high-level expression of transgenic TCRs competes with endogenous TCRs for expression in developing T cells, leading to lower numbers of T cells with an endogenous TCR (Attridge and Walker, 2014). This accounts for the observed Treg cell paucity as the frequency of T cells potentially cross-reacting with thymically presented self-peptides is diminished, which, apart from immediate negative selection, restricts their conversion into thymus-derived(t)Treg (Attridge and Walker, 2014).

To test the T cell functionality in TCR-DR1 mice, we first studied their allergen-specific T cell responses *in vitro*. Incubation with the cognate Art v 1_25–36_-peptide or the recombinant Art v 1 protein but not a immunodominant control peptide of another potent aero-allergen (Bet v 1_142–153_ of the major birch pollen allergen, which otherwise very efficiently stimulates T cells equipped with a Bet v 1_142–153_-specific TCR (Neunkirchner et al., 2011)), or medium alone, induced proliferation (Fig. S3**, B** and **C**) and secretion of a Th0-like cytokine pattern in TCR-DR1 splenocytes (Fig. S2B), similar to the original human T cell clone (Leb et al., 2008). Allergen-specific proliferation was dose-dependent, with maximum and half-maximum proliferation reached at 125 nM and 57.2 ± 14.8 nM, respectively, with full-length rArt v 1 (Fig. 1**C**), and 1.5 μM and 0.284 ± 0.079 μM, respectively, with Art v 1_25–36_ peptide (Fig. S3D). Upon incubation with the immunodominant Art v 1_25–36_ peptide, >35% of the CD3^+^CD4^+^ T cells entered proliferation (Fig. S4) while no proliferation was observed using a control peptide (Fig. S4, **B** and **C**). Collectively, these data are indicative of proper selection and function of the allergen-specific TCR on the HLA-DR1 background and provide evidence for immunogenic processing and presentation of Art v 1_25–36_ peptide in an HLA-DR1-restricted manner to naïve allergen-specific T cells, even with full length Art v 1 protein applied.

### 3.2 Acute airway hyperreactivity in TCR-DR1 mice upon sole respiratory exposure to aqueous mugwort pollen extract in the absence of adjuvants

**Fig. 2:**
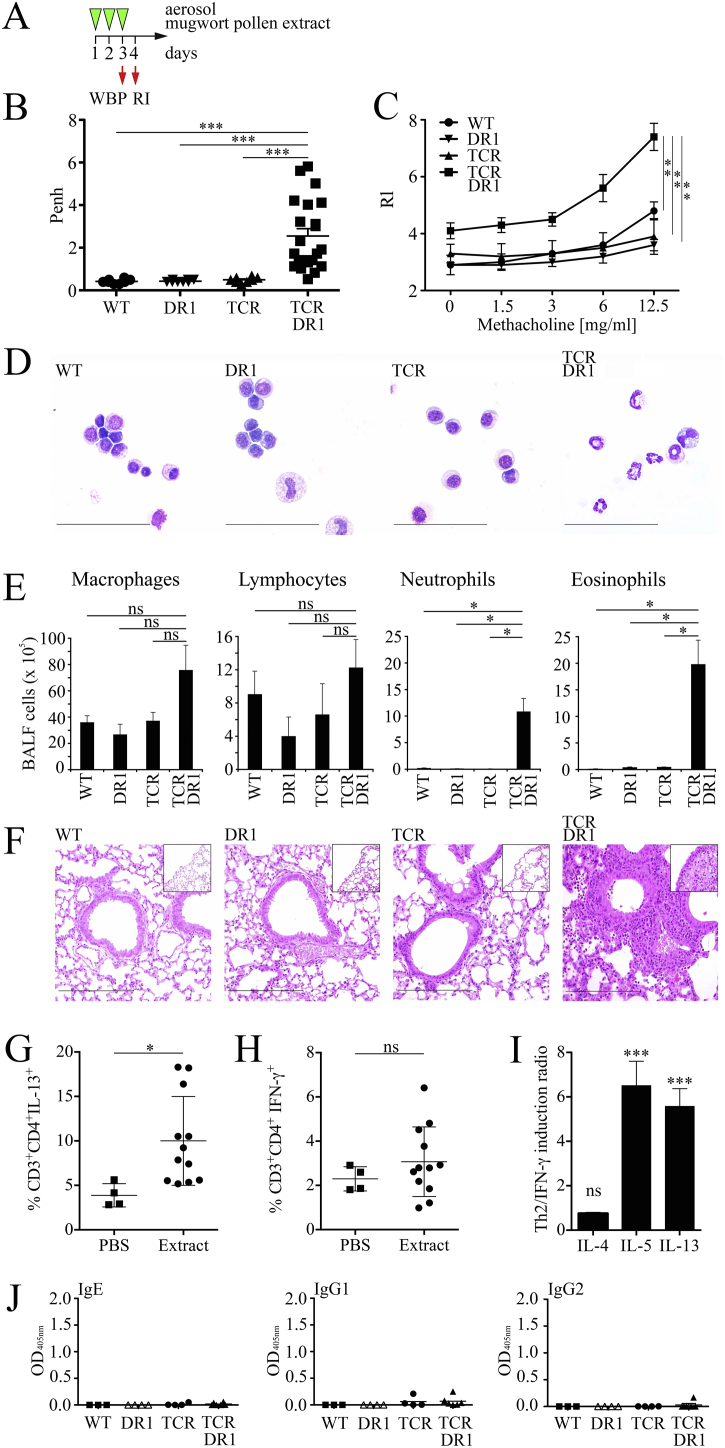
Rapid induction in TCR-DR1 mice of airway hyperreactivity and inflammation in the absence of allergen-specific antibody production. **A,** Experimental design for exposure of mice (WT, DR1, TCR, or TCR-DR1) to 1% nebulized mugwort-pollen extract (3 mg extract/mouse) in a whole body plethysmograph (Buxco) for 16 min on three consecutive days (green arrow heads). Allergen-specific airway hyperreactivity was determined at the end of the 3rd exposure on day 3 (WBP, whole body plethysmography), lung resistance (Rl) on day 4. **B**, Shown are mean enhanced pause (Penh) values for each mouse (individual symbols). **C**, Lung resistance (RI) was determined on day 4 (Buxco, Finepoint Software). **D**, and **E**, Shown are cytospin preparations (modified Wright-Giemsa stain, size bars 50 μm) and mean numbers of BALF cells. **F**, Shown are H&E stainings of lung tissues (insets peripheral lung tissues) analyzed by light microscopy, size bars 100 μm. **G**, and **H**, Shown are the percentages of CD3^+^CD4^+^IL13^+^ and CD3^+^CD4^+^IFN-γ^+^ T cells in lung suspensions of PBS or mugwort extract challenged TCR-DR1 mice. Each symbol represents an individual mouse. **I**, Cytokine ratios of PHA-restimulated lung cells comparing fold-induction of IL-4, IL-5 and IL-13 with IFN-γ. **J**, Allergen-specific serum Ig levels (day 4). Symbols represent individual mice. Data show the summary (*B, E*) or are representative (*C, D, F, G*) of 12 (except 22 for TCR-DR1) mice per group of four independent experiments (*B, D, E, F, H, I, J*), or three mice per group of three independent experiments (*C*), or four mice per group (except three for WT) of two independent experiments (*J*), or 12 (except 4 for PBS) mice per group of two independent experiments (*G, H, I*). *p < .05, **p < .01, ***p < .001; ns, not significant; ANOVA and Tukey's multiple comparison test or Student's *t*-test (*H*, *I*), respectively.

A previous study performed with the major cat dander allergen Fel d 1 in HLA-DR1 transgenic mice suggested that two intraperitoneal (i.p.) systemic priming steps with Al(OH)_3_-adjuvanted allergen followed by three intranasal challenges with aqueous allergen extract are required to induce lung inflammation and affect lung function (Campbell et al., 2009). However, in humans sensitization to aero-allergens exclusively occurs by inhalation *via* the airways. Moreover, it is well known that adjuvants, such as the frequently used Al(OH)_3_, apart from activating the inflammasome in a NRLP3-dependent manner (Eisenbarth et al., 2008) also lead to structural rearrangements in co-administered proteins that not only deviate the immune response to different components found in allergen preparations but also change epitope specificities (Hansen et al., 2011). Therefore, to closely mimic the human situation and to avoid non-physiological prime/boost protocols, we evaluated AHR and lung pathology upon allergen-specific sensitization and challenge with adjuvant-free aerosolized mugwort pollen extract (Fig. 2**A**). TCR-DR1 mice presented with significantly increased AHR after three aerosol exposures (day 3) using whole body plethysmography (WBP, Fig. 2**B**), which was confirmed using invasive lung resistance measurements (Rl) on day 4 (Fig. 2**C**). Control (WT, DR1 or TCR mice) mice showed no significant changes in lung function. Analyses of bronchoalveolar lavage fluids (BALF) of exposed mice revealed increased leukocyte numbers in TCR-DR1 mice (TCR-DR1: 12.5 ± 6.7 × 10^6^), as compared to control mice (WT: 4.3 ± 2.8 × 10^6^; DR1 3.2 ± 1.6 × 10^6^; TCR: 4.4 ± 1.1 × 10^6^) (Fig. 2**, D** and **E**), caused by neutrophils and eosinophils (Fig. 2E). Numbers of macrophages and lymphocytes were increased as well but did not reach statistical significance (Fig. 2E). Moreover, lungs of TCR-DR1 mice were severely inflamed, presenting with grade 3 cellular infiltrates (Fig. 2**F**) dominated by eosinophilic granulocytes (Fig. S5A). Moreover, CD4^+^ T cells and CD45R/B220^+^ B cells accumulated in inflamed lung tissues in TCR-DR1 mice (Fig. S5**, B** and **C**). Similar results were obtained by flow cytometric analyses of lung digests of TCR-DR1 but not control mice (Fig. S5D). Further gating proved that BALF of control mice contained mostly CD11c^+^LY6C/G^+^SiglecF^+^ alveolar macrophages, while BALF of TCR-DR1 mice was dominated instead by CD11c^−^LY6C/G^+^SiglecF^−^ neutrophils and CD11c^−^LY6C/G^low^SiglecF^+^ eosinophils (not shown).^.^ Notably, exposure of TCR-DR1 mice to mugwort extract significantly increased numbers of CD3^+^CD4^+^IL-13^+^ but not CD3^+^CD4^+^IFN-γ^+^ T cells in lung tissues (Fig. 2**, G** and **H**). Restimulation of lung cell homogenates revealed both increased Th1 and Th2 cytokine levels. However, the fold induction of the Th2 cytokines IL-5 and IL-13 was found to be higher than for the Th1 cytokine IFN-γ, resulting in significantly elevated IL-5/IFN-γ and IL-13/IFN-γ ratios of 6.5 ± 1.1 and 5.6 ± 0.8, respectively (Fig. 2**I**).

Due to the short time frame of the protocol, no relevant Art v 1-specific immunoglobulin production was detected (Fig. 2**J**). Apart from the respiratory route, the T cell-dependent, allergen-specific response was also observed upon injecting aqueous mugwort pollen extract into the hind footpads of mice, with significant swelling exclusively in TCR-DR1 mice within 72 h (Fig. S6). This implies that the model may also be suitable to study the IgE-independent aspects of allergic skin reactions, such as those frequently observed in the majority of atopic dermatitis patients in which anti-IgE treatment is ineffective (Wang et al., 2016). In addition, the model will allow to study in detail the pathomechanisms of the “skin-lung crosstalk”, which has shown, that intradermal introduction of immunodominant T cell epitopes of major allergens may lead to late-phase asthma exacerbations (Haselden et al., 1999).

**Fig. 3:**
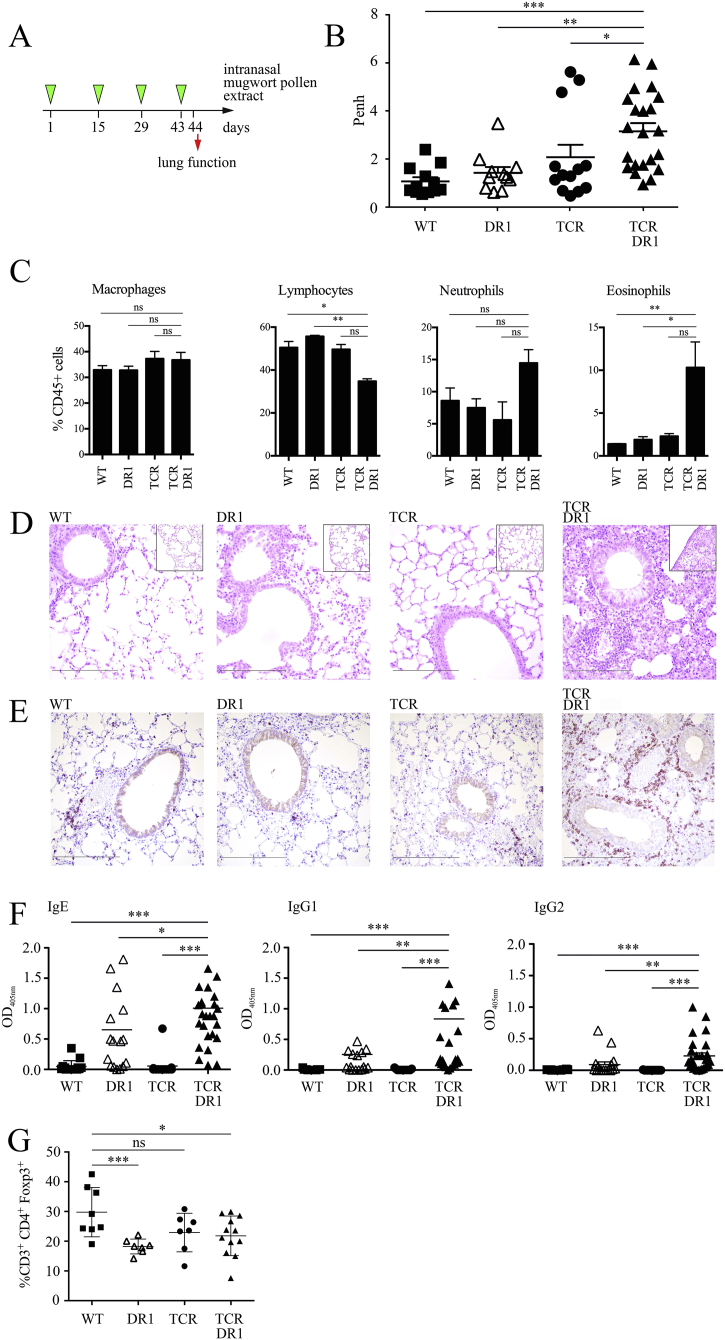
Severe lung pathology in TCR-DR1 mice upon intranasal exposure to aqueous mugwort pollen extract. **A,** Experimental design: Intranasal exposure of mice (WT, DR1, TCR, or TCR-DR1) to four doses of aqueous mugwort pollen extract (450 μg in 20 μl) at bi-weekly intervals. **B,** Allergen-specific airway hyperreactivity was measured 24 h after the last exposure by inhalation of 1% nebulized aqueous mugwort pollen extract aerosol and the mean enhanced pause (Penh) was determined by unrestricted whole body plethysmography (Buxco). Symbols represent individual mice. **C,** Shown are flow cytometric analyses of BALF cells gated on the indicated cell populations. **D,** H&E and **E,** immunohistochemical CD3 stainings of lung tissues analyzed by light microscopy, size bars 100 μm. **F,** Allergen-specific serum Ig levels (day 44). **G,** Percentages of CD3^+^CD4^+^Foxp3^+^ positive T cells after exposure. Data show the summary (*B*, *F*, *G*) or are representative (*C*, *D*, *E*) of 12 (for wild-type (WT), 11 for DR1, 13 for TCR and 22 for TCR-DR1) mice per group of three independent experiments (*B*–*E*) or 15 (for WT, 17 for DR1, 13 for TCR and 28 for TCR-DR1) mice per group of five independent experiments (*F*), or 12 for TCR-DR1 (except 8 for WT and 7 each for DR1 and TCR, respectively) mice of two independent experiments (*G*). *p < .05, **p < .01, ***, *p* < .001. ANOVA and Tukey's multiple comparison test.

TCR-DR1 mice undergoing chronic i.n. exposure (four times bi-weekly) to adjuvant-free mugwort pollen extract showed similarly impaired lung function (AHR) as determined by whole body and invasive measurements (Fig. 3**, A** and **B** and Fig. S7). Notably, i.n. sensitization and challenge led to pronounced BALF eosinophilia and neutrophilia in TCR-DR1 mice (Fig. 3**C**). Histopathological examination revealed that lungs of i.n. sensitized TCR-DR1 mice, similar to those of aerosol challenged mice, presented with a high degree of inflammation (Fig. 3**D**), which was absent in the other mouse lines tested. Besides eosinophils, CD3^+^ T cells represented a major lung infiltrating population in TCR-DR1 but not control mice (Fig. 3**E**). With this protocol, TCR-DR1 mice also developed allergen-specific IgE (Fig. 3**F**), which were functionally active in RBL degranulation assays (not shown). Of note, also DR1 mice generated allergen-specific immunoglobulins, however, at lower percentages. This demonstrates that in genetically susceptible mice a high penetrance of the allergen-specific, IgE producing phenotype is reached only when efficient T cell help is provided, *i.e.,* when the preexisting allergen-specific T cell repertoire is expanded such as in TCR-DR1 mice. Interestingly, the paucity of lung CD3^+^CD4^+^Foxp3^+^ T cells in TCR-DR1 mice remained present in these long-term/chronically exposed mice (Fig. 3**G**).

### 3.3 IL-2-αIL-2 complexes expand Tregs, alleviate airway hyperreactivity and keep allergen-specific IgE levels low

Elevated allergen-specific Th cell precursor frequencies and low numbers of allergen-specific Treg, as seen in our TCR-DR1 mouse model, represent a typical constellation observed in individuals at risk for the development of allergic diseases (Hartl et al., 2007; Ling et al., 2004). Therefore, our mouse model not only closely mimics allergic individuals but also offers the chance to investigate the effect of elevating Treg numbers as a prophylactic and therapeutic approach for allergy control.

**Fig. 4:**
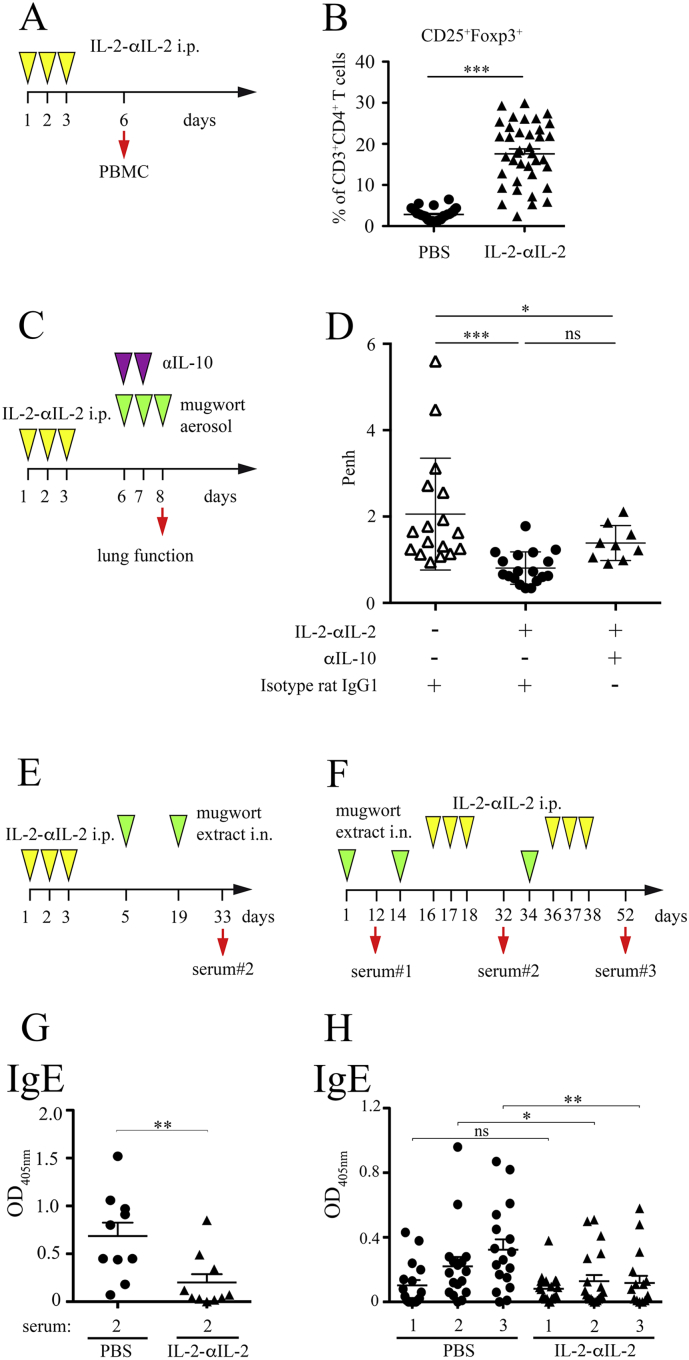
IL-2-αIL-2 mAb-dependent *in vivo* expansion of Treg cells mitigates subsequent sensitization to mugwort extract. **A,** Scheme for induction and phenotypic analysis of Treg cells induced by IL-2-αIL-2 (1 μg mIL-2 complexed to 5 μg JES6-1 mAb). **B,** Summary of CD3^+^CD4^+^CD25^+^ PB T cells co-expressing Foxp3 of sham (PBS) or IL-2-αIL-2 treated TCR-DR1 mice obtained on day 6. Symbols represent individual mice. **C,** Scheme to test the influence of IL-2-αIL-2 complexes on AHR in the presence or absence of αIL-10 or control mAb TCR-DR1 mice. **D,** IL-2-αIL-2 complex treatment leads to reduced AHR upon aerosol sensitization and challenge with 1% nebulized mugwort pollen extract (3 mg/mouse) of TCR-DR1 mice, which is only modestly reversed by αIL-10 mAb *in vivo*. Data show the summary (*B, D*) of 33 IL-2-αIL-2 and 30 PBS treated mice per group (*B*) and 18 (for IL-2-αIL-2 complex and isotype control mAb, 17 for isotype control mAb, and 9 for IL-2-αIL-2 complex and αIL-10 mAb) treated mice (*D*) that were analyzed in five (*D*) and four (except one for αIL-10 mAb treated mice) (*D*) independent experiments. **E,** Scheme for the prophylactic treatment of mice with IL-2-αIL-2 complexes and **F,** corresponding allergen-specific serum Ig levels. **G,** Scheme for the therapeutic treatment of mice with IL-2-αIL-2 complexes and **H,** corresponding allergen-specific serum Ig levels. Serum IgE levels of mice treated according to (*E*) or (*F*) with IL-2-αIL-2 complexes or PBS used as control substance. Art v 1-specific IgE levels determined by ELISA and expressed in arbitrary units (OD 405 nm) are shown. Symbols represent individual mice. Data show the summary of 10 (*G*) or 20 (*H*) (except 18 for PBS treated) mice per group analyzed in two (*G*) or three (*H*) independent experiments. *p < .05, **p < .01, ***p < .001, ns, not significant. Student's *t*-test and ANOVA, followed by Tukey's multiple comparison test.

To rebalance the Teff:Treg-ratio, we here used systemic i.p. administration of IL-2 complexed to the IL-2 specific antibody JES6–1 on three consecutive days (Fig. 4**A**), known to efficiently expand Treg *in vivo* (Boyman et al., 2006). Treatment of TCR-DR1 mice with IL-2-αIL-2 mAb complexes induced significant increases of CD3^+^CD4^+^CD25^+^ T cells co-expressing Foxp3^+^ (6.2-fold, range 2.8 ± 1.3% to 17.6 ± 7.4%) (Fig. 4**B**), CD39^+^ (8.1-fold, range 2.1 ± 1.5% to 17.2 ± 7.9%) and KLRG1^+^ (7.1-fold, range 0.2 ± 0.2% to 1.6 ± 0.7%) (Fig. S8**, A** to **D**) that peaked on day 6 (not shown). Under these conditions, no CD4^+^ Teff cell and only a modest NK cell expansion, as shown previously (Boyman et al., 2006), could be observed, excluding putative effects of non-complexed, free IL-2. Of relevance to the allergic phenotype, IL-2-αIL-2 complex-induced Tregs were functional, conferring protection to IgE-independent, allergen-induced AHR in TCR-DR1 mice (Fig. 4**, C** and **D**) and inhibiting the proliferation of allergen-activated Teff cells *in vitro* (Fig. S9). This indicated that elevated numbers of Treg protect from allergen sensitization. Blocking of IL-10, a potent anti-inflammatory cytokine produced by Tregs (de Vries, 1995), on days 6 and 7 directly before allergen exposure only modestly reverted the IL-2-αIL-2 complex-induced alleviation of AHR (Fig. 4**D**). Similarly, αIL-10 mAbs did not reduce the suppressive effect of IL-2-αIL-2 mAb complex-generated Treg *in vitro* (Fig. S9).

Another protective mechanism aimed at in allergen-specific immunotherapy is to prevent/reduce the production of allergen-specific IgE and/or promote the induction of allergen-specific blocking IgG antibodies (Freidl et al., 2017) that neutralize allergens and deliver negative regulatory signals (Burton et al., 2014). Notably, TCR-DR1 mice pretreated with IL-2-αIL-2 mAb complexes before mugwort sensitization (Fig. 4**E**) presented with significantly reduced Art v 1-specific serum IgE levels when compared to sham-treated mice (Fig. 4**G**). Therapeutic treatment with IL-2-αIL-2 complexes inhibited a further increase of allergen-specific IgE levels (Fig. 4**, F** and **H**) while maintaining IgG2 (Fig. S10**, A** to **D**). Allergen-specific IgG2 antibodies represent Th1-dependent correlates of protective antibodies known to either neutralize allergens before they can reach IgE-armed mast cells in tissues (Flicker and Valenta, 2003) or delivering FcγRIIb-dependent, negative-regulatory signals (Burton et al., 2014).

## 4 Discussion

We established a novel humanized mouse model to study the mechanisms of allergic sensitization and to answer the hitherto unanswered question why certain individuals develop allergy against certain allergens. In the past, several murine models attempting to mimic allergic asthma have been developed. Some were based on murine transgenic TCRs with specificity for model allergens (*e.g.,* chicken ovalbumin) (Kappler et al., 1981; Murphy et al., 1990) or *bona fide* human-relevant allergens (*e.g.,* Der p 1 (Coquet et al., 2015; Jarman et al., 2005)), some are based on the expression of human MHC (*e.g.*, HLA-DR1 (Campbell et al., 2009)). Furthermore, numerous regimens for their sensitization and challenge have been applied (Zosky and Sly, 2007). Our model is unique because we were able to compare mice which are double transgenic for the TCR specific for a major human-relevant allergen (*i.e.,* Art v 1) and the human restriction element DR1 to mice that were transgenic only for the TCR or DR1, and the corresponding wildtype mice. We intentionally bred the three transgenic mouse lines on the C57BL6J background to avoid deficiency in mouse MHC class II, which otherwise might have led to skewing of the murine CD4^+^ T cell repertoire. The four mouse lines allowed us to investigate the contribution of MHC-dependent recognition of allergen-specific T cell epitopes, the impact of the balance of allergen-specific Teff:Treg cells and the role of natural antigenic exposure to allergic sensitization, the key event leading to allergic disease.

Our data strongly support a predominant role of the genetic restriction of allergen presentation in allergic sensitization, because single DR1 tg mice were able to produce allergen-specific IgE after chronic allergen exposure whereas single TCR tg and WT mice did not. Thus, our data confirm at the experimental animal level the genetic susceptibility for allergy to a major allergen source (*i.e.*, mugwort pollen) observed in the human population (Jahn-Schmid et al., 2005). Expression of the human HLA-DRA*01:01/HLA-DRB1*01:01 heterodimer in transgenic mice in the absence of the co-expression of the human, allergen-specific TCR elicited allergen-specific IgE production, however, in fewer animals and only with modest levels of lung eosinophilia and neutrophilia and without gross alterations of lung function. The fact that the TCR single transgenic mice did not develop any signs of allergy under identical conditions of sensitization and challenge suggests genetic control of allergen presentation as being the primary and superior factor in allergic sensitization. Notably, previous genome wide association studies (GWAS) have shown significant associations between the presence of certain HLA class II gene polymorphisms, including critical residues in their peptide binding region and the susceptibility to environmental and food allergens on the population level (Hinds et al., 2013; Hong et al., 2015; Martino et al., 2017). While the almost exclusive HLA-DR1 restriction of mugwort allergy represents a unique case, restrictor analysis tool for epitopes(RATE)-testing (Paul et al., 2015) for other major allergen sources has revealed that a (small) group (but definitely not other) of alleles might predispose, *e.g*., for allergen-specific T cell responses to allergens such as *Ambrosia* (Pham et al., 2016). Thus, the conclusions made here within a highly defined biological system and centered on one major allergen, can most likely be generalized and extended to other allergens, such as those present in cat dander (Hinds et al., 2013) and food (Hong et al., 2015; Martino et al., 2017).

Cognate T cell help and regulation, however, is important as well in modulating the allergic immune response. Allergen-specific immunotherapy (AIT) studies helped to understand the beneficial contribution of Tregs to allergy control (Akdis and Akdis, 2014), showing that increased Treg cell numbers and/or function in individuals susceptible to allergic diseases help controlling allergic immune responses. Tregs were present but clearly diminished in numbers in TCR-DR1 mice. This Treg cell paucity is not unique to our model and has been described previously for other TCR tg models and seems to reflect intraclonal competition between transgenic and endogenously formed TCRs in T cells during thymic selection (Attridge and Walker, 2014). Notably, Treg cell paucity (Hartl et al., 2007; Ling et al., 2004) together with elevated precursor frequencies of allergen-specific CD4^+^ effector T cells induced herein by TCR transgenesis, are both highly reminiscent of the constellation found in human individuals suffering from respiratory allergies. Thus, balancing/correcting the Teff:Treg-ratio might help controlling the allergic phenotype.

Our strategy of exposing four different lines of mice to mugwort pollen extract allowed us to dissect the individual contributions to sensitization and development of allergic disease of T cells and restriction elements in otherwise “un-manipulated” mice. Since the frequency of allergen-specific T cells in TCR-DR1 mice is high (approximately 60%), the two major branches of adaptive allergen-specific responses, *i.e.,* allergen-presentation and T cell activation *versus* allergen-specific IgE production could be studied in sequence, using the acute *versus* the chronic exposure model. Thus, it is appropriate to investigate sensitization in mouse strains with high allergen-specific T cell precursor frequencies, because this allows to directly study the consequences of allergen exposure *via* the natural routes, *i.e.,* the airways in the case of aeroallergens in the absence of adjuvants, systemic priming or intratracheal instillation of non-physiological concentrations of allergens. Similar studies, in which mice with high allergen-specific T cell precursor frequencies have been sensitized and challenged, have been performed in the past to study different aspects of AHR (Jarman et al., 2005; Knott et al., 2000) or to prime house dust mite major allergen-specific T cells to more closely characterize the role of B cells during allergen presentation (Dullaers et al., 2017).

We here used an improved IL-2 formulation based on IL-2-αIL-2 immune-complexes leading to increased bioavailability of IL-2 (Votavova et al., 2014) and its more efficient targeting to the high-affinity IL-2R containing CD25 (Boyman et al., 2006). The expanded CD25^+^Foxp3^+^ T cells co-expressed CD39, the prototype ecto-nucleoside triphosphate diphosphohydrolase responsible for the generation of AMP from ATP, which together with CD73 forms the basis for the provision of immunosuppressive adenosine by Treg (Borsellino et al., 2007). Moreover, a fraction of the *in vivo* expanded Treg co-expressed the killer-cell lectin-like receptor G1, KLRG1, marking highly-activated Treg preferentially residing in mucosal tissues (Cheng et al., 2012). Notably, IL-2-αIL-2 complexes led to a clear-cut expansion of allergen-specific Treg expressing the clonotypic TCR (not shown). We found that Treg expansion by systemic administration of IL-2-αIL-2 complexes (Boyman et al., 2006) was capable of protecting the genetically susceptible TCR-DR1 mice from the consequences of natural allergen exposure, *i.e.,* sensitization and prototypic organ pathology in both prophylactic and therapeutic settings. This is the first humanized allergy model in which, upon natural induction, IL-2-αIL-2 complexes could alleviate both sensitization and disease. Mechanistically, we found that IL-2-αIL-2 complex-induced peripheral(p)Treg function did not solely depend on IL-10. Blocking IL-10 only modestly de-repressed the Treg-induced amelioration of AHR (Fig. 4*D*). In the future, our model will thus allow us to interrogate other putative mechanisms utilized by IL-2-αIL-2 immune-complex-induced Treg, including adenosine and TGF-β1 among others (Schmetterer et al., 2011b) as well as the longevity of their suppressive function in a systematic manner.

Our data identify i) genetic restriction of allergen presentation as key factor for allergic sensitization and ii) highlight the role of the Teff:Treg balance in the control of the allergic immune response. However, we did not find evidence that allergens, that rapidly dissociate from inhaled particles (*e.g.*, pollen) and become soluble in aqueous solutions, more readily escape Treg-mediated suppression and thus drive allergen-specific Teff responses and allergic sensitization as was claimed recently (Bacher et al., 2016). In fact, natural allergen exposure to aqueous (*i.e*., soluble) allergen extract neither led to significant allergic sensitization to any of the other soluble mugwort pollen antigens present in the mugwort pollen extract (data not shown) nor to lung pathology in the absence of the restricting MHC, *i.e.,* HLA-DR1. These data argue against the hypothesis that the composition of the allergen (particulate *versus* soluble) is a *primary factor* dictating allergic sensitization *versus* protection (Bacher et al., 2016). While the results obtained in the four mouse strains under study were clear-cut, it cannot be excluded at this point that different principles apply for the human (outbred) population, as claimed (Bacher et al., 2016). However, the latter model also does not explain why individuals living in a certain geographical area and who are exposed to a similar panel of soluble antigens do not develop uniform allergic sensitization (Siroux et al., 2017).

So far, associations between HLA class II alleles and sensitization to distinct allergen sources have been described only for a restricted number of allergens, which puts mugwort allergy, with its very strong association with the HLA-DRB1*01 allele, into an exceptional position. While the strong HLA-DRB1*0101 association helped to establish a robust biological model from which clear-cut experimental conclusions can be drawn, it also represents a potential limitation when it comes to comparisons with other allergen sources and putatively governed by less prominent HLA class II restrictions. To address this potential limitation the authors are in the process of establishing human TCR tg mice with birch pollen reactivity.

Conceivably, other important factors may contribute to allergic sensitization. Besides the confounding role of infections (Rubner et al., 2017; Stein et al., 1999), barrier-relevant mutations (Rodriguez et al., 2009) and pollutants (Eckl-Dorna et al., 2010; Parnia et al., 2002), it had been suggested that certain allergens are efficient sensitizers due to their enzymatic activities (Gough et al., 1999). Dose, route of encounter as well as adjuvants (*e.g.,* lipid mediators) also play an important role (Valenta, 2002). The presence of Th2-driving adjuvants in pollen has been reported previously (Traidl-Hoffmann et al., 2005) and may, in fact, explain why the TCR-DR1 transgenic mice developed a predominant Th2 response *in vivo* upon exposure *via* the airways, *i.e.,* the major target organ of aeroallergens, although the Art v 1-specific activation of *ex vivo* stimulated splenocytes of TCR-DR1 mice did not induce an *a priori* bias towards either of the Th cell subsets.

Lung resident type 2 innate lymphoid cells (ILC2) have been shown to function as a potential early source for IL-5 and IL-13, and are supposed to contribute to allergen-independent AHR and asthma (Kim et al., 2012). ILC2s may play a role in modulating the allergen-specific immune response towards Th2 in TCR-DR1 mice. However, using the natural conditions applied here they were unable to drive sensitization in WT and single TCR transgenic mice in the absence of optimal genetic restriction (HLA-DR1), although WT mice can be efficiently sensitized when mugwort pollen extract is applied i.p. systemically (not shown). The fact that single TCR tg mice, in contrast to TCR-DR1 mice, could not be sensitized at all speaks against the possibility that the mere introduction of the TCR transgene has led to numerical and/or functionally relevant alterations in ILC2s between the two mouse lines, since the TCR-DR1 line is a direct descendent of the TCR single tg line.

In future, our model will be extremely useful for the development of new preventive and therapeutic strategies for allergy because it closely mimics human disease. The TCR-DR1 mice are specific for a clinically highly-relevant major mugwort pollen allergen, Art v 1, derived from *Artemisia vulgaris* pollen. *Artemisia*-sensitization is detectable in 16.8% of individuals transferred for skin-prick testing to allergy clinics in Europe, and affects up to 44.3% and 28.3% of such individuals in Hungary or Denmark, respectively (Heinzerling et al., 2009). Symptoms of *Artemisia vulgaris* (mugwort) allergy comprise mild (*i.e.,* hay fever) but also very severe forms of allergy (*i.e.,* asthma), which are typically observed during late summer and beginning of fall (Torio et al., 2003). The TCR-DR1 mice can be sensitized, similar to humans, *via* the respiratory route and will help to promote the development and evaluation of novel therapeutic management strategies for allergic diseases. In particular, future cell transfer studies will allow us to unequivocally determine i) the nature of the antigen presenting cell type responsible for priming and establishment of memory responses of mugwort-specific T cells, ii) the contribution of skewing and/or pool-size of involved T helper cell populations under these circumstances and iii) the influence of targeted therapies based on, *e.g.,* small molecule inhibitors impacting on the relevant innate and adaptive immune cells (Tauber and Pickl, 2017). In a first attempt along those lines, we have shown here that expansion of Tregs by IL-2-αIL-2 mAb complexes represents such a promising strategy in both prophylactic and therapeutic settings. Moreover, our model offers ample opportunities for additional translational studies. For instance, novel human-relevant modes of clinical tolerance induction (Smole et al., 2017) and active immune deviation can be studied in our model. These might rely on B cell epitope-based vaccines (Zieglmayer et al., 2016), T cell epitope-based tolerance induction (Valenta et al., 2012) and altered peptide ligand(APL)-centered immune deviation strategies (Candia et al., 2016) or non-allergenic formulations of allergens, *e.g.,* shielded within virus-like nanoparticles (Kueng et al., 2010).

In summary, we demonstrate the primary importance of HLA-restricted allergen-presentation and the balance between allergen-specific Teff:Treg cell precursor frequencies for eliciting and propagating allergies. This murine model will also open new avenues for the various fields of basic allergy research as well as to study clinical tolerance induction.

## Funding sources

Austrian Science Fund (SFB F4609-B13, F4605-B13, F4610-B13, SFB F1807-B13, DK W1248-B30 and FWF 20011-B13), Biomay AG, and Christian Doppler Research Association (Christian Doppler Laboratory for Immunmodulation), Austria. Funders did not have any role in study design, data collection, data analysis, interpretation and writing of the report.

## Conflicts of interest

WFP holds stocks of Biomay AG and receives honoraria from Novartis. RV has received research grants from Biomay AG and Viravaxx, Austria and serves as a consultant for Biomay AG, Viravaxx.

## Appendix A Supplementary data

Supplementary material Image 1
