# Supplementary material for: Genetic restriction of antigen-presentation dictates allergic sensitization and disease in humanized mice
Source: eBioMedicine. 2018 Apr 5;31:66–78. doi: 10.1016/j.ebiom.2018.04.001 (PMC6014064; doi:10.1016/j.ebiom.2018.04.001)
Supplement: Supplementary file 1 — Supplementary material [file mmc1.pdf]

Genetic restriction of antigen-presentation dictates allergic sensitization and disease in humanized mice

Alina Neunkirchner [a,b], Bernhard Kratzer [a,b,*], Cordula Köhler [a,b,*], Ursula Smole [b], Lukas F. Mager [b], Klaus G. Schmetterer [b,c], Doris Trapin [b], Victoria Leb-Reichl [b], Edward Rosloniec [d,e,f], Ronald Naumann [g], Lukas Kenner [h,i,j], Beatrice Jahn-Schmid [k], Barbara Bohle,[a,k] , Rudolf Valenta [k], and Winfried F. Pickl [a,b, **]

Short title: Humanized allergy mice

[a] Christian Doppler Laboratory for Immunomodulation, 1090 Vienna, Austria
[b] Institute of Immunology, Medical University of Vienna, 1090 Vienna, Austria
[c] Department of Laboratory Medicine, Medical University of Vienna, 1090 Vienna, Austria
[d] Department of Medicine, University of Tennessee Health Science Center, Memphis, 38163 TN, USA.
[e] Memphis Veterans Affairs Medical Center, 38104 TN, USA
[f] Department of Pathology, University of Tennessee Health Science Center, Memphis, 38163 TN, USA
[g] Max Planck Institute for Molecular Cell Biology and Genetics, 01307 Dresden, Germany
[h] Department of Laboratory Animal Pathology, Medical University of Vienna, 1090 Vienna, Austria
[i] Department of Laboratory Animal Pathology, University of Veterinary Medicine Vienna, 1210 Vienna, Austria
[j] Ludwig Boltzmann Institute for Cancer Research, Vienna, Austria
[k] Department of Pathophysiology and Allergy Research, Medical University of Vienna, 1090 Vienna, Austria

* contributed equally to the work

** corresponding author: Winfried F. Pickl, MD, Institute of Immunology, Center for Pathophysiology, Infectiology and Immunology, Medical University of Vienna, Lazarettgasse 19, 1090 Vienna, Austria. Phone: (+431) 40160 33245. Fax: (+431) 40160 933245. Email address: winfried.pickl@meduniwien.ac.at. ORCID ID: orcid.org/0000-0003-0430-4952

Appendix A. Supplementary Data

Fig. S1. Illustration of the generation of TCR transgenic targeting constructs using genomic TCR cassette vectors.

Fig. S2. Expression of HLA-DR1 on dendritic cells and elaboration of a mixed set of cytokines of upon activation TCR-DR1 splenocytes.

Fig. S3. Treg paucity and antigen-specific activation of T lymphocytes in the four mouse lines under study.

Fig S4. Art v 1-specific proliferation of $CD4^+$ T cells as determined by CPD eFluor® 450 dilution.

Fig. S5. Preferential accumulation of inflammatory cells in the lungs of sole mugwort aerosol challenged TCR-DR1 mice.

Fig. S6. Footpad swelling upon mugwort extract exposure in TCR-DR1 mice.

Fig. S7. Intranasal exposure to mugwort pollen extract of TCR-DR1 mice leads to an increase in lung resistance.

Fig. S8. IL-2-αIL-2 complexes expand Treg *in vivo*.

Fig. S9. Effects of blocking αIL-10 mAbs on *in vitro* division of allergen-specific T cells.

Fig. S10. Reduction of allergen-specific immunoglobulin levels in mice treated with IL-2-αIL-2 complexes.

Table S1. List of monoclonal antibodies used for flow cytometric analyses of spleens, thymi and peripheral blood leukocytes

Table S2. Total cell numbers and percentages of indicated cell types in spleen, thymus and peripheral blood of WT, DR1, TCR and TCR-DR1 transgenic mice.

Supplementary Materials:

FIGURE S1

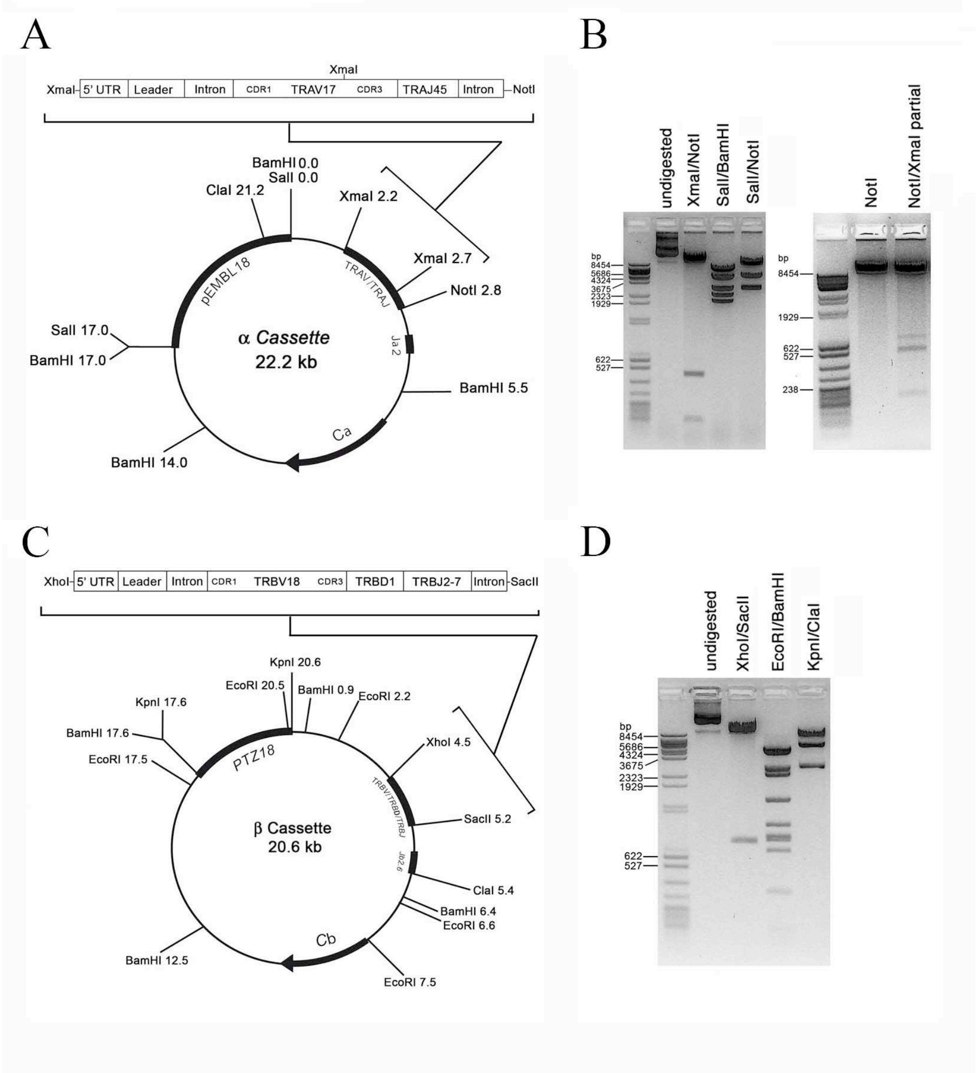

Fig. S1. Illustration of the generation of TCR transgenic targeting constructs using genomic TCR cassette vectors. A, Schematic depiction of the TCR alpha transgenic expression vector. The vector (22.2 kb) consists of 2.2 kb of genomic DNA upstream of the murine TCR alpha leader, variable and joining sequence (0.6 kb) and 14.2 kb of downstream homologous murine TCR sequences including the Jα2, the Cα exon and the 3' enhancer elements according to Kouskoff et al. (Kouskoff et al., 1995) and based on Bluthmann et al. (Bluthmann et al., 1988). The position of the 5.2 kb prokaryotic vector sequences (pEMBL18) are indicated. Unique restriction sites, *Xma I* and *Not I*, were used for inserting the genomic human TCR alpha leader, variable and joining sequences, while *Sal I* sites were used to remove the prokaryotic plasmid sequences to linearize the entire TCR gene (17.1 kb). **B,** *Xma I/Not I*-digested vector DNA was used to identify properly inserted TCR variable sequences

and comparisons between the undigested (lane 1) *Xma I/Not I*-digested (lane 2), *Sal I/BamH I*-digested (lane 3) and *Sal I/Not I*-vector (lane 4) and vector DNA partially digested with *Not I* and *Xma I/Not I* are shown. **C,** Schematic depiction of the TCR beta transgenic expression vector. The vector (20.6 kb) consists of 4.5 kb of genomic DNA upstream of the murine TCR beta leader, variable and joining sequence (0.8 kb) and 12.4 kb of downstream homologous murine TCR beta sequences including the Jβ2.6, Cβ exon and 3' enhancer elements according to Kouskoff et al.(Kouskoff et al., 1995) and based on Bluthmann et al. (Uematsu et al., 1988). The position of the 2.9 kb prokaryotic vector sequences (PTZ18) are indicated. The unique restriction sites *Xho I* and *Sac II* for inserting the respective genomic human TCR beta leader, variable, diversity and joining sequences are indicated as well as the *Kpn I* sites used to remove the prokaryotic plasmid sequences for linearization of the entire TCR beta gene (17.6 kb). **D**, *Xho I/Sac II*-digested vector DNA was used to identify properly inserted TCR variable sequences (lane 2) and was compared to undigested vector DNA (lane 1), digest profile of Eco RI/Bam HI (lane 3) or Kpn I/Cla I (lane 4) digestion. Positions of DNA size markers (mixture of λ*Bst EII* digest and pBR322 *Msp I* digest) are indicated.

FIGURE S2

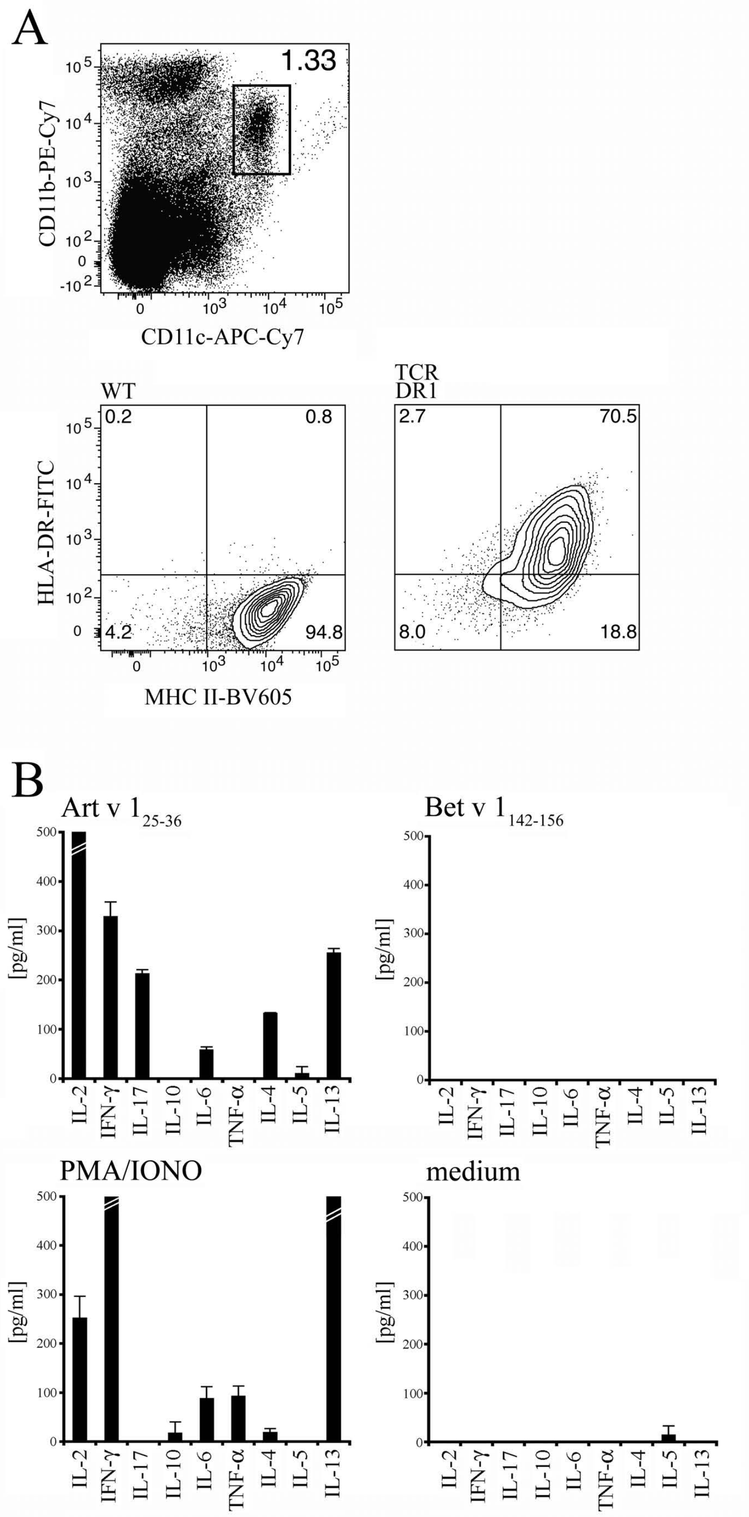

Fig. S2. Expression of HLA-DR1 on dendritic cells and elaboration of a mixed set of cytokines of upon activation TCR-DR1 splenocytes. A, Shown is HLA-DR1 expression by flow cytometry on CD11b^{+}CD11c^{+} dendritic cells of spleens of TCR-

DR1 and WT mice. **B**, Splenocytes of TCR-DR1 mice were incubated with Art v $1_{25\text{-}36}$ peptide, Bet v $1_{142\text{-}153}$ peptide, medium alone or PMA plus ionomycin. Art v $1_{25\text{-}36}$ peptide but neither Bet v $1_{142\text{-}153}$-peptide nor medium alone induced production of high-levels (> 500 pg/ml per $2x10^5$ cells /well) of IL-2, intermediate levels (499-200 pg/ml) of IFN-γ, IL-13 and IL-17 and low levels (199-10 pg/ml) of IL-4 and IL-6, while no indication for TNF-α, IL-5 and IL-10 secretion was observed. PMA/ionomycin incubation, used as positive control, induced clear-cut levels (>10 pg/ml) of IL-2, IFN-γ, IL-10, IL-6, TNF-α, IL-4 and IL-13. Data are representative of two independently performed experiments.

FIGURE S3

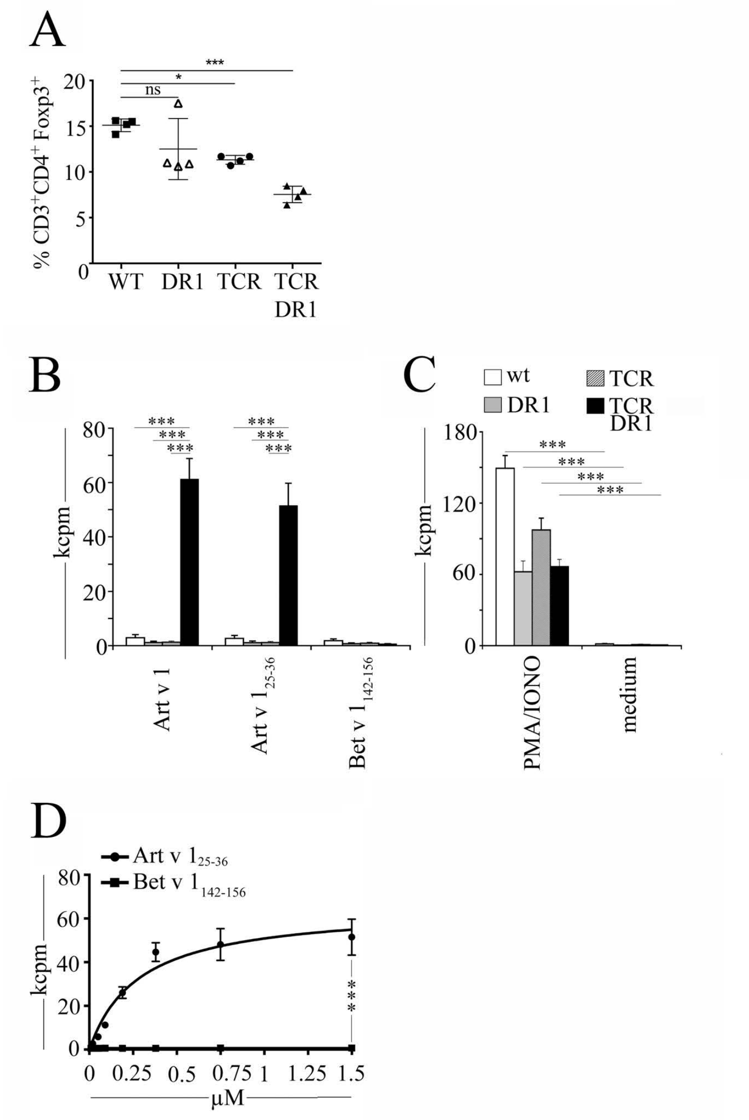

Fig. S3. Treg paucity and antigen-specific activation of T lymphocytes in the four mouse lines under study.

A, Shows the percentages of $CD3^+CD4^+Foxp3^+$ cells in spleens of WT, DR1, TCR and TCR-DR1 mice. Each symbol represents an individual mouse. **B,** Shown is the proliferation of splenocytes (2 x 10^5/well) from WT, DR1, TCR and TCR-DR1 mice incubated with full length rArt v 1-protein (3 *µ*M), Art v $1_{25\text{-}36}$-peptide or negative control Bet v $1_{142\text{-}153}$-peptide (10 *µ*M) *in vitro*. **C,** Shows additional positive (PMA/ionomycin) and negative (medium) controls. **D,** Shown is the dose dependent proliferation of splenocytes from TCR-DR1 mice upon incubation with Art v $1_{25\text{-}36}$-peptide in comparison to negative control Bet v $1_{142\text{-}153}$-peptide. kcpm indicates kilo counts per minute of incorporated [^{3}H]-thymidine. Data shown are mean values ± SEM of triplicate cultures of one experiment, which is representative of four independent experiments. *** $p < 0.001$, Kruskal-Wallis test and Mann-Whitney-U-test followed by *post hoc* Bonferroni correction.

FIGURE S4

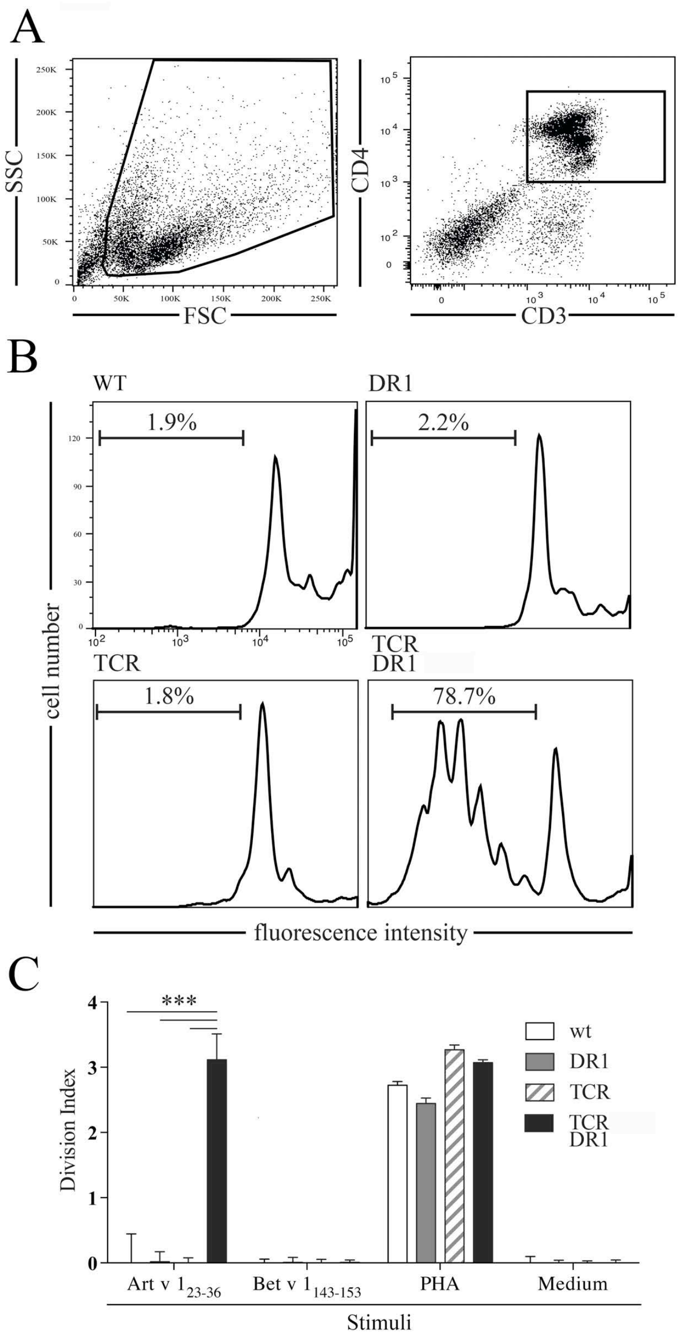

Fig S4. Art v 1-specific proliferation of $CD4^+$ T cells as determined by CPD eFluor® 450 dilution. Flow cytometry analysis showing proliferation of CPD eFluor® 450-labeled $CD3^+CD4^+$TCR T cells, left either untreated (medium) or incubated with Art v $1_{25\text{-}36}$-peptide, Bet v $1_{142\text{-}153}$-peptide or PHA for 96 hours. **A**, Dot plots show gating strategy, **B**, Histograms show cellular proliferation. Markers show cells, which underwent proliferation. **C**, Shown are division indices of CPD-labeled $CD3^+CD4^+$ T cells calculated according to established methods as described (Asquith et al., 2006). Data are representative (*A, B*) or show the summary (*C*) of three independent

experiments. ***, $p < 0.001$; Kruskal-Wallis test, followed by post hoc Bonferroni correction.

FIGURE S5

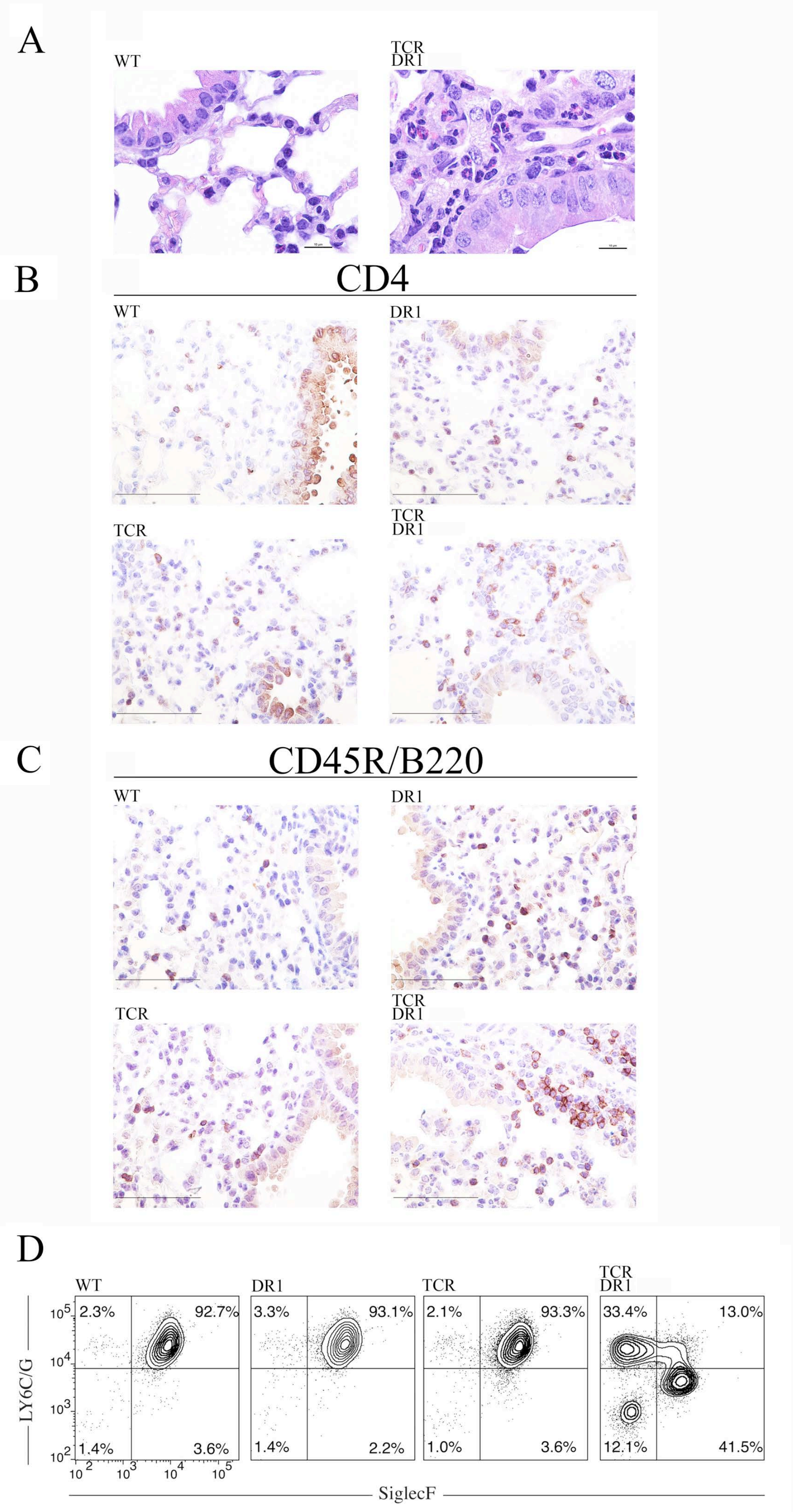
A
WT
TCR DR1
B
CD4
WT
DR1
TCR
TCR DR1
C
CD45R/B220
WT
DR1
TCR
TCR DR1
D
WT
DR1
TCR
TCR DR1
2.3%
92.7%
1.4%
3.6%
3.3%
93.1%
1.4%
2.2%
2.1%
93.3%
1.0%
3.6%
33.4%
13.0%
12.1%
41.5%
LY6C/G
SiglecF

Fig. S5. Preferential accumulation of inflammatory cells in the lungs of sole mugwort aerosol challenged TCR-DR1 mice. A, Shown are HE-stained lung sections at high-power magnification, arrowheads show eosinophilic granulocytes. **B,** and **C,** Shown are immunohistochemical CD4 (b) and CD45R/B220 (c) stainings of lung tissues analyzed by light microscopy, size bars 50 μm. Data are representative of three experiments performed. **D,** Shown are two parameter contour plots of flow cytometric analyses of lung tissues of WT, DR1, TCR and TCR-DR1 mice, respectively. Only lung homogenates of TCR-DR1 mice show the appearance of high percentages of LY6C/G^{+}SiglecF^{neg} neutrophils and of LY6C/G^{dim}SiglecF^{+} eosinophils. Numbers show cells in respective quadrants.

FIGURE S6

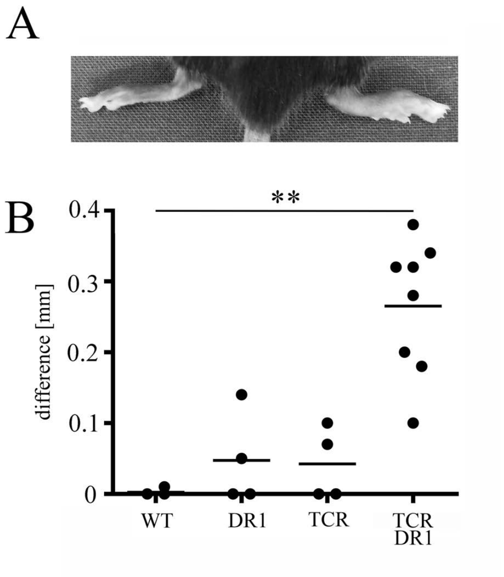

Fig. S6. Footpad swelling upon mugwort extract exposure in TCR-DR1 mice. WT, DR1, TCR and TCR-DR1 mice were injected with 25 *μ*l of allergen extract preparation, corresponding to 375 *μ*g whole protein content intradermally into the right hind footpad. The contralateral left hinder food pad was challenged with PBS as control. **A,** Shown is a representative photograph of the hind footpads of a challenged TCR-DR1 mouse. **B,** Shown is the footpad swelling before and after challenge with mugwort extract using a digital thickness gauge. The changes in footpad swelling were determined as follows: Footpad swelling (mm) = footpad thickness after allergen provocation - footpad thickness before allergen provocation. Each symbol shows an individual mouse 72 hours after injection. Eight mice in the TCR-DR1 group were compared to four mice in all other groups. Data are representative of three independent experiments. **, $p < 0.01$; Kruskal-Wallis test followed by post hoc Bonferroni correction

FIGURE S7

A

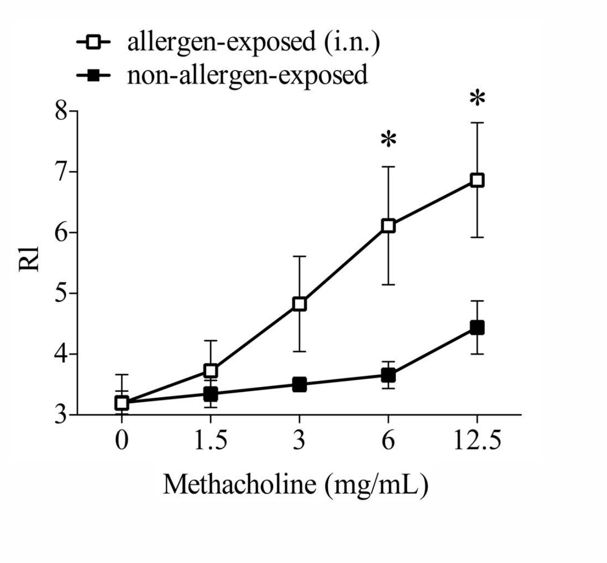

Fig. S7. Intranasal exposure to mugwort pollen extract of TCR-DR1 mice leads to an increase in lung resistance. Mice were exposed four times to 450 µg mugwort pollen extract in PBS intranasally in bi-weekly intervals according to the scheme shown in FIG 3, A. Shown is lung resistance (Rl) determined one day after the last exposure on day 44 (Buxco, Finepoint software) by inhalation of increasing doses of methacholine as indicated. Every symbol indicates mean lung resistance levels of each group of mice (allergen exposed versus non-allergen exposed); vertical lines show the standard error of mean (SEM). Data are representative of five allergen exposed and four non-allergen exposed mice. * $p < 0.05$, allergen-exposed compared to non-allergen-exposed mice by Mann-Whitney-U-test.

FIGURE S8

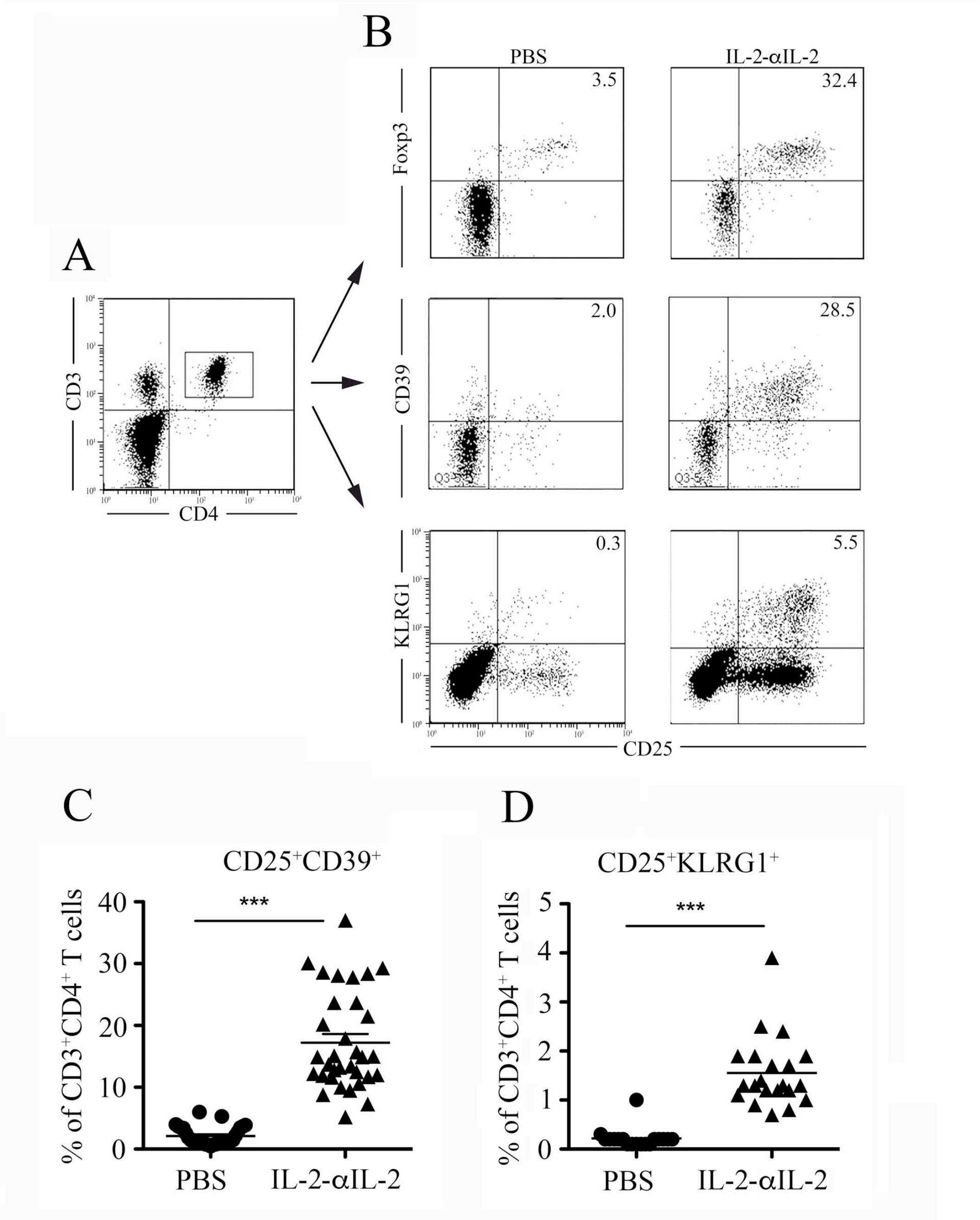

Fig. S8. IL-2-αIL-2 complexes expand Treg *in vivo*. A, Gating strategy for analysis of Treg cells expanded by IL-2-αIL-2 complexes **B,** phenotypic appearance and summary of $CD3^{+}CD4^{+}CD25^{+}$ PB T cells co-expressing **C**, CD39 or **D**, KLRG1, respectively, of sham (PBS) or IL-2-αIL-2 treated TCR-DR1 mice obtained on day 6. Numbers indicate the percentages of cells in the double positive quadrant. Each symbol represents an individual mouse. Data are representative (A, B) or show the summary (C, D) of stainings for 33 IL-2-αIL-2 (except 21 for CD25/KLRG1) and 30 PBS treated (except 19 for CD25/KLRG1) mice per group that were analyzed in five independent experiments. ***, $p < 0.001$, Unpaired two-tailed Student's t-test (d) and ANOVA, followed by Tukey's multiple comparison test.

FIGURE S9

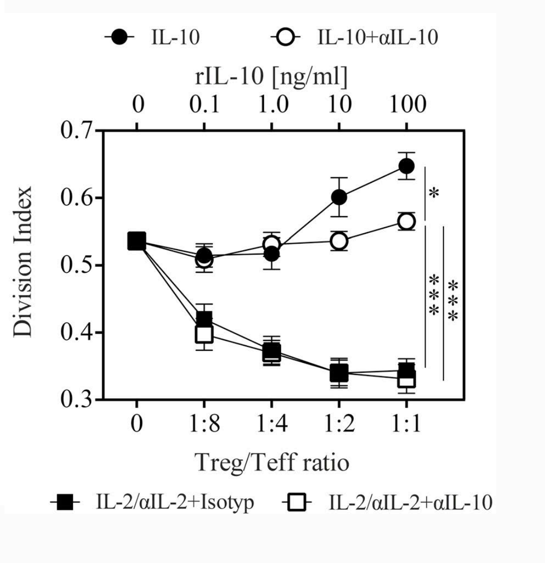

Fig. S9. Effects of blocking αIL-10 mAbs on *in vitro* division of allergen-specific T cells. FACS-sorted $CD3^+CD4^+CD25^-$ effector T cells from allergen-naive TCR-DR1 mice were incubated with FACS-sorted $CD3^+CD4^+CD25^{bright}$ Treg of IL-2-α-IL-2 complex treated TCR-DR1 mice at the indicated ratios. Either isotype control or αIL-10 mAb (20μg/ml) were added to the culture together with 1 μM of Art v 1_{23-36} peptide along with DC2.4 $DR1^+$ dendritic cells, serving as APCs. Proliferation of effector T cells labelled with violet-CPD is represented as division index obtained after 96 hours. Instead of Treg, soluble rIL-10 in the presence of αIL-10 or isotype control mAb added to the cultures served as control. Shown are the mean ± SEM division indices pooled from two independent experiments. * $p < 0.05$, *** $p < 0.001$. One-way ANOVA followed by Tukey's multiple comparison test.

FIGURE S10

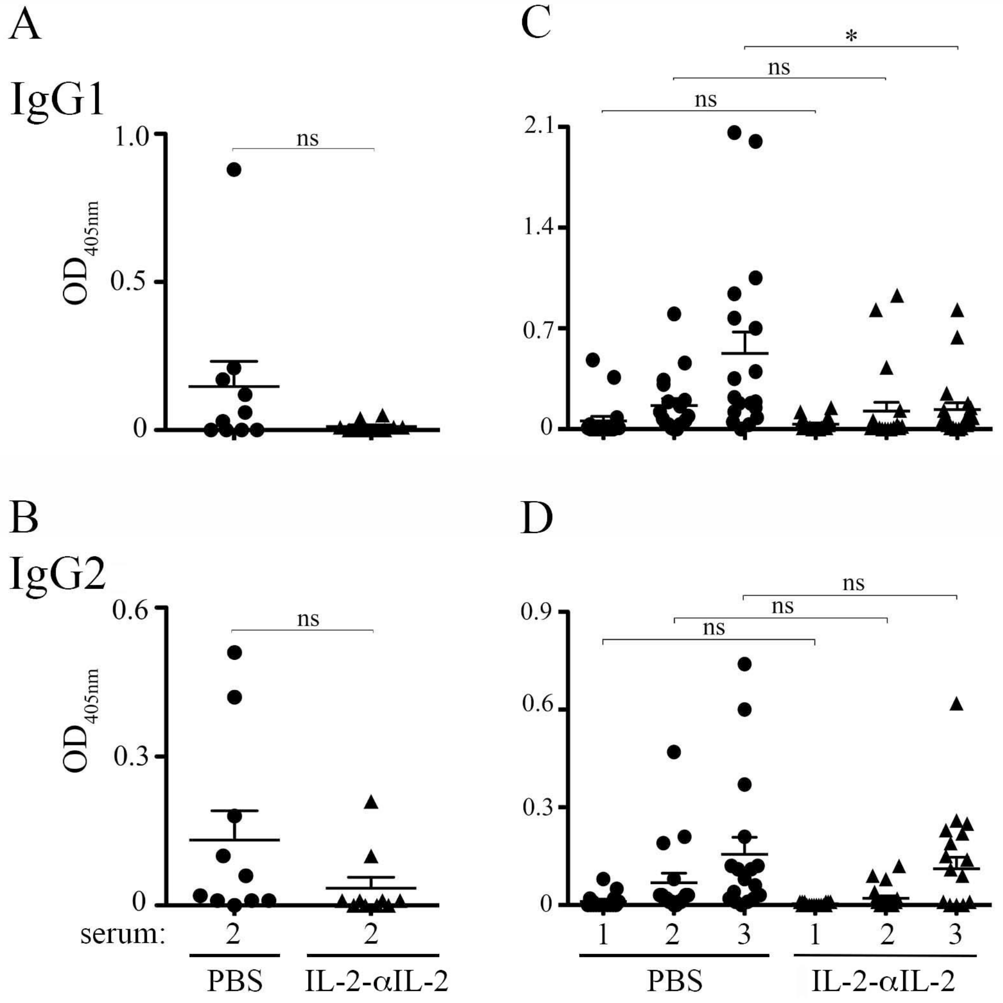

Fig. S10. Reduction of allergen-specific immunoglobulin levels in mice treated with IL-2-aIL-2 complexes.

Allergen-specific serum immunoglobulin **A,** and **C**, IgG1 and **B,** and **D**, IgG2 levels upon prophylactic (A, B) or therapeutic (C, D) treatment of mice with IL-2-αIL-2 complexes. Serum immunoglobulin levels of mice treated with PBS were used as control. Shown are Art v 1-specific IgG1 and IgG2 levels determined by ELISA and expressed in arbitrary units (OD 405nm). Each symbol represents an individual mouse. Data show the summary of 10 (A, B) or 20 (except 18 for PBS treated) (C, D) mice per group analyzed in two (A, B) or three (C, D) independent experiments. ns, not significant, * $p < 0.05$, ANOVA, followed by Student's t-test.

Table S1. List of monoclonal antibodies

Table S1A. List of monoclonal antibodies used for flow cytometric analyses of spleens, thymi and peripheral blood leukocytes

Specificity	Clone name	Species	conjugated to	Source
CD3	500A2	hamster	FITC	eBioscience, San Diego, CA, USA
CD3	17A2	rat	eFluor450	eBioscience, San Diego, CA, USA
CD4	RM4-5	rat	eV450	eBioscience, San Diego, CA, USA
CD4	RM4-5	mouse	APC	eBioscience, San Diego, CA, USA
CD8	5H10	rat	APC	Invitrogen, Camarillo, CA, USA
CD14	rmC5-3	rat	PE	Becton Dickinson, Palo, Alto, CA
CD45R/B220	RA3-6B2	rat	FITC	eBioscience, San Diego, CA, USA
CD45R/B220	RA3-6B2	rat	PB	eBioscience, San Diego, CA, USA
CD90.2 (Thy1.2)	53-2.1	rat	APC	eBioscience, San Diego, CA, USA
Ly6C+6G	RB6-8C5	rat	FITC	Becton Dickinson, Palo, Alto, CA
HLA-CII	L243	mouse	FITC	BioLegend, San Diego, CA, USA
HLA-CII	L243	mouse	PerCP	BioLegend, San Diego, CA, USA
TRBV18	BA62.6	mouse	PE	Immunotech, Marseille, France
TCRγ/δ	GL3	hamster	FITC	BioLegend, San Diego, CA, USA
control	eBio299Arm	hamster	FITC	eBioscience, San Diego, CA, USA
control	P3.6.2.8.1	mouse	PE	eBioscience, San Diego, CA, USA
control	MOPC-21	mouse	PerCp	BioLegend, San Diego, CA, USA
control	MOPC-21	rat	APC	BioLegend, San Diego, CA, USA
control	RTK2758	rat	PO/PB	BioLegend, San Diego, CA, USA

Table S1B. List of monoclonal antibodies used for flow cytometric analysis BALF

Specificity	Clone name	Species	conjugated to	Source
CD16/CD32	2.4G2	rat	-	Becton Dickinson, Palo, Alto, CA
CD45	30-F11	rat	Alexa Fluor 700	Becton Dickinson, Palo, Alto, CA
CD3	500A2	hamster	PE	eBioscience, San Diego, CA, USA
CD4	L3T4	rat	Pe-Cy7	Becton Dickinson, Palo, Alto, CA
CD11c	HL3	hamster	APC-Cy7	Becton Dickinson, Palo, Alto, CA

CD45R/B220	RA3-6B2	rat	PB	BioLegend, San Diego, CA, USA
CD19	1D3	rat	V450	Becton Dickinson, Palo, Alto, CA
CCR3	83103	rat	Alexa Fluor 647	Becton Dickinson, Palo, Alto, CA
Siglec-F	E50-2440	rat	Alexa Fluor 647	Becton Dickinson, Palo, Alto, CA
Ly6C+6G	RB6-8C5	rat	FITC	Becton Dickinson, Palo, Alto, CA
Ly6C+6G	RB6-8C5	rat	Pacific Orange	Molecular Probes
CD117	2B8	rat	PE-CF594	Becton Dickinson, Palo, Alto, CA
IgE	R35-72	rat	FITC	Becton Dickinson, Palo, Alto, CA
NK-1.1	PK136	rat	PerCP-Cy5.5	Becton Dickinson, Palo, Alto, CA
HLA-DR	L243	mouse	PerCP	BioLegend, San Diego, CA, USA
MHC I-A/I-E	M5/114.15.2	rat	PerCP	BioLegend, San Diego, CA, USA
control	eBio299Arm	hamster	FITC	eBioscience, San Diego, CA, USA
control	P3.6.2.8.1	mouse	PE	eBioscience, San Diego, CA, USA
control	MOPC-21	mouse	PerCp	BioLegend, San Diego, CA, USA
control	MOPC-21	rat	APC	BioLegend, San Diego, CA, USA
control	RTK2758	rat	PO/PB	BioLegend, San Diego, CA, USA

Table S1C. List of monoclonal antibodies used for flow cytometric analyses of Foxp3 expression in peripheral blood

Specificity	Clone name	Species	conjugated to	Source
CD3	500A2	hamster	PerCP-Cy5.5	eBioscience, San Diego, CA, USA
CD4	RM4-5	rat	BV510	Becton Dickinson, Palo, Alto, CA
CD25	PC61	rat	BV421	Becton Dickinson, Palo, Alto, CA
CD39	24DMS1	rat	PE-Cy7	eBioscience, San Diego, CA, USA
Foxp3	FJK-16s	rat	APC	eBioscience, San Diego, CA, USA
KLRG1	2F1	hamster	FITC	eBioscience, San Diego, CA, USA
TRBV18	BA62.6	mouse	PE	Immunotech, Marseille, France
control	eBio299Arm	hamster	PerCP-Cy5.5	eBioscience, San Diego, CA, USA
control	P3.6.2.8.1	mouse	PE	eBioscience, San Diego, CA, USA
control	eBio299Arm	mouse	FITC	BioLegend, San Diego, CA, USA
control	MOPC-21	rat	APC	BioLegend, San Diego, CA, USA
control	RTK2758	rat	PE-Cy7	BioLegend, San Diego, CA, USA
control	R35-95	rat	BV510	Becton Dickinson, Palo, Alto, CA
control	R35-95	rat	BV421	Becton Dickinson, Palo, Alto, CA

Table S1D. List of monoclonal antibodies used for flow cytometric analyses of transcription factors and cytokines in lung homogenates

Specificity	Clone name	Species	conjugated to	Source
CD3	500A2	hamster	FITC	eBioscience, San Diego, CA, USA
CD4	RM4-5	rat	Alexa Fluor 700	Becton Dickinson, Palo Alto, CA
T-bet	eBio4B10	mouse	PerCP-Cy5.5	eBioscience, San Diego, CA, USA
GATA3	L50-823	mouse	PE-Cy7	Becton Dickinson, Palo Alto, CA
Foxp3	FJK-16s	rat	APC	eBioscience, San Diego, CA, USA
IFN- γ	XMG1.2	rat	APC	eBioscience, San Diego, CA, USA
IL-13	eBio13A	rat	PE	eBioscience, San Diego, CA, USA
control	eBio299Arm	hamster	FITC	eBioscience, San Diego, CA, USA
control	R35-95	rat	Alexa Fluor 700	Becton Dickinson, Palo Alto, CA,
control	P3.6.2.8.1	mouse	PerCP-Cy5.5	eBioscience, San Diego, CA, USA
control	MOPC-21	mouse	PE-Cy7	Becton Dickinson, Palo Alto, CA
control	RTK2758	rat	APC	BioLegend, San Diego, CA, USA
control	R35-95	rat	PE	Becton Dickinson, Palo Alto, CA

Abbreviations: APC, allophycocyanine; PE, phycoerythrin; Cy, cyanine; PerCP, peridinin chlorophyll protein; FITC, fluorescein isothiocyanate, PO/PB, pacific orange/pacific blue, BV, brilliant violet

Table S1E. List of monoclonal antibodies used for cytometric bead array-based analyses of secreted cytokines

Specificity	Clone name	Species	conjugated to	Source
IL-4	11B11	rat		eBioscience, San Diego, CA, USA
IL-5	TRFK5	rat		eBioscience, San Diego, CA, USA
IL-13	eBio13A	rat		eBioscience, San Diego, CA, USA
IFN-γ	AN-18	rat		eBioscience, San Diego, CA, USA
IL-4	BVD6-24G2	rat	biotin	eBioscience, San Diego, CA, USA
IL-5	TRFK4	rat	biotin	eBioscience, San Diego, CA, USA
IL-13	eBio1316H	rat	biotin	eBioscience, San Diego, CA, USA
IFN-γ	R4-6A2	rat	biotin	eBioscience, San Diego, CA, USA
Streptavidin			PE	eBioscience, San Diego, CA, USA

Table S2. Total cell numbers and percentages of indicated cell types in spleen, thymus and peripheral blood of WT, DR1, TCR and TCR-DR1 transgenic mice.

		Spleen		Thymus		Peripheral Blood	
		Percentages	Absolute numbers (*10^6 /organ)	Percentag es	Absolute numbers (*10^6 /organ)	Percentag es	Absolute numbers (*10^3 /mm^3)
**T cells**							
CD3	*WT*	25.1±3.8	24.4±9.8	96.7±0.7	147.1±33.6	27.0±4.4	3.6±0.2
(TRBV18+)		0.2± 0.2	0.2±0.1	0.0±0.0	0.3±0.0	0.0±0.0	0.2±0.0
CD3	*DR1*	24.9± 5.5	20.7±4.1	96.0±1.5	135.6±53.7	35.8±3.6	3.3±0.8
(TRBV18+)		0.2± 0.1	0.1±0	0.0±0.0	0.3±0.0	0.0±0.0	0.0±0.0
CD3	*TCR*	17.3± 2.5	20.7± 2.0	95.9±0.5	72.8±30.9	19.3±4.7	1.9±0.5
(TRBV18+)		7.6±3.7	9.0± 4.1	12.7±2.7	25.9±4.3	13.9±4.0	1.4±0.5
CD3	*TCR/DR1*	19.5±4.4	20.4±6.6	96.1±1.2	103.1±30.3	24.0±3.2	3.0±0.5
(TRBV18+)		14.6± 5.0	14.9±4.6	11.3±1.5	63.1±4.8	17.8±2.9	2.2±0.4
CD4	*WT*	14.4± 2.8	14.0± 5.8	9.8±0.9	15.0±4.0	14.1±2.8	2.0±0.1
(TRBV18+)		0.1± 0	0.1±0.0	0.1±0.1	0.2±0.1	0.0±0.0	0.0±0
CD4	*DR1*	19.1± 4.9	15.7±3.1	17.3±2.8	23.2±5.8	26.2±2.7	2.4±0.6
(TRBV18+)		0.1± 0.1	0.1±0.1	0.2±0.2	0.2±0.1	0.0±0.0	0.0±0.0
CD4	*TCR*	11.3± 1.4	13.5±0.4	8.9±2.7	6.9±4.4	11.6±2.6	1.1±0.3
(TRBV18+)		4.8±3.4	6.3±3.9	5.0±3.1	4.0±2.7	8.0±4.3	0.8±0.4
CD4	*TCR/DR1*	17.5±4.0	18.2±5.4	20.3±4.8	22.0±9.2	19.5±3.0	2.4±0.5
(TRBV18+)		9.1±7	13.6±7.1	11.3±5.6	18.7±6.4	12.1±8.1	1.6±1.1
CD8	*WT*	8.7± 1.9	8.7±4.3	4.2±0.4	6.4±1.8	8.8±1.4	1.1±0.2
(TRBV18+)		0.0± 0.0	0.0±0.0	0.0±0.0	0.0±0.0	0.0±0.0	0.0±0.0
CD8	*DR1*	3.9± 1.1	3.2±0.8	3.4±0.6	4.7±1.8	6.1±0.3	0.6±0.1
(TRBV18+)		0.0± 0.0	0.0±0.0	0.0±0.0	0.0±0.0	0.0±0.0	0.0±0.0
CD8	*TCR*	4.0± 1.6	4.8±1.8	5.0±2.2	3.8±2.0	4.7±1.5	0.5±0.2
(TRBV18+)		1.1± 0.8	1.3±0.8	1.3±0.5	1.0±0.6	3.5±1.3	0.3±0.1
CD8	*TCR/DR1*	0.8± 0.5	0.8±0.4	2.8±0.9	3.1±1.6	0.8±0.5	0.1±0.1
(TRBV18+)		0.4± 0.3	0.4±0.2	1.9±0.8	2.2±1.3	0.6±0.3	0.1±0.0
**B cells**							
CD45R	*WT*	52.3±5.9	49.3±13.3	0.8±0.8	1.2±1.4	45.1±4.8	6.2±1.8
*HLA-DRB1*01:01+*		0.1±0.1	0.1±0.1	0.0±0.0	0.0±0.0	0.1±0.0	0±0
CD45R	*DR1*	37.0±2.5	32.7±11.8	0.2±0.1	0.2±0.1	26.2±2.4	2.4±0.5
*HLA-DRB1*01:01+*		29.1±1.3	25.3±8.0	0.2±0.1	0.1±0.1	22.4±1.7	2.0±0.4
CD45R	*TCR*	69.7±13.5	69.7±13.5	2.2±0.7	1.6±0.8	46.2±3.8	4.5±0.8
*HLA-DRB1*01:01+*		0.2±0.0	0.2±0.0	0.0±0.0	0.0±0.0	0.2±0.0	0±0
CD45R	*TCR/DR1*	48.9±26.5	48.9±26.5	0.6±0.2	0.7±0.2	38.7±4.0	4.9±0.9
*HLA-DRB1*01:01+*		40.6±23.6	40.6±23.6	0.6±0.1	0.6±0.1	33.5±2.9	4.2±0.7
**Lymphocytes**							
	WT	80.0±4.9	76.7±25.0	96.1±0.9	145.9±32.0	71.6±3.6	9.8±2.3
	DR1	75.0±2.3	65.7±22.1	95.0±1.4	134.0±52.9	62.9±1.8	5.7±1.1
	TCR	81.2±1.3	98.1±14.4	94.4±1.8	71.4±29.9	46.2±3.8	5.9±1.0
	TCR/DR1	73.9±2.5	79.8±32.5	95.9±0.7	102.9±30.1	56.9±4.4	7.1±0.6
**Monocytes**							
CD14	*WT*	0.1±0.0	0.1±0.0	0.0±0.0	0.0±0.0	0.2±0	0.0±0
*HLA-DRB1*01:01+*		0.0±0.0	0.0±0.0	0.0±0.0	0.0±0.0	0,0±0	0.0±0
CD14	*DR1*	0.1±0.0	0.1±0.0	0.0±0.0	0.0±0.0	0.2±0	0.0±0
*HLA-DRB1*01:01+*		0.0±0.0	0.0±0.0	0.0±0.0	0.0±0.0	0,0±0	0.0±0
CD14	*TCR*	0.1±0.0	0.1±0.0	0.0±0.0	0.0±0.0	0.2±0	0.0±0
*HLA-DRB1*01:01+*		0.0±0.0	0.0±0.0	0.0±0.0	0.0±0.0	0,0±0	0.0±0
CD14	*TCR/DR1*	0.1±0.0	0.1±0.0	0.0±0.0	0.0±0.0	0.2±0	0.0±0
*HLA-DRB1*01:01+*		0.0±0.0	0.0±0.0	0.0±0.0	0.0±0.0	0.1±0	0.0±0
**Granulocytes**							
Ly6C+Ly6G+	*WT*	9.6±2.2	9.4±4.7	2.1±0.3	3.3±1.0	19.9±3.4	2.7±0.4
	DR1	12.9±2.0	11.6±4.5	3.5±1.0	4.7±2.1	28.5±4.3	2.6±0.7
	TCR	8.2±1.0	9.8±1.8	3.4±1.1	2.8±2.0	29.7±6.4	2.8±0.6
	TCR/DR1	9.8±1.7	10.6±4.8	2.2±0.2	2.4±0.9	32.5±1.5	4.1±0.6
**Leukocytes**	*WT*		86.6±27.4		149.2±31.0		12.4±2.3
	DR1		77.7±24.5		138.2±49.9		8.4±1.5
	TCR		108.6±15.0		74.1±29.4		8.8±1.4
	TCR/DR1		92.1±34.5		105.0±28.6		11.4±1.1

Grey numbers indicate numbers and percentage of TCR Vβ18^{+} or HLA-DR1+ cells, respectively. Data show the summary of five mice per group analyzed in five independent experiments. Mean ± S.D. is shown.
